# Engineered *Aspergillus niger* enables high-yield heterologous kojic acid production

**DOI:** 10.1016/j.synbio.2026.03.003

**Published:** 2026-03-22

**Authors:** Jiao Liu, Bingbing Xie, Fengna Gao, Jing Kang, Hao Liu

**Affiliations:** Key Laboratory of Industrial Fermentation Microbiology, Ministry of Education, Tianjin Key Laboratory of Industrial Microbiology, College of Biotechnology, Tianjin University of Science and Technology, Tianjin Economic-Technological Development Area (TEDA), No. 9, 13th Avenue, Tianjin, 300457, PR China

**Keywords:** Kojic acid, *Aspergillus niger*, Metabolic engineering, Microbial cell factory

## Abstract

Industrial-scale production of kojic acid (KA)—valued in cosmetics, food, and pharma—has been hindered by limited metabolic engineering strategies for native producers (e.g., *Aspergillus oryzae*), which largely rely on random mutagenesis. To address this, *Aspergillus niger* was engineered as a heterologous host. A combinatorial strategy was employed, which included multi-copy integration of His-tagged *kojA*, overexpression of heterologous transporter *AokojT*, deletion of repressor *nrkC*, amplification of global regulator *laeA*, and expression of *Vitreoscilla* hemoglobin *vgb* enhancing oxygen utilization. This integrated approach progressively increased KA titer to 38 g/L in shake flasks. Medium optimization achieved stable pH (∼5.5) without buffering agents, elevating production to 50 g/L. Scale-up in a bioreactor yielded 56 g/L KA (0.58 g/g yield; 8.0 g/L/day productivity), demonstrating strong industrial potential. This engineered strain provides a high-yield platform and offers a transferable metabolic engineering framework for KA overproduction in both heterologous and native fungal producers.

## 1 Introduction

Kojic acid (KA), chemically defined as 5-hydroxy-2-(hydroxymethyl)-4-pyrone, is a versatile secondary metabolite valued for its antioxidant, tyrosinase-inhibitory, and antimicrobial properties. These attributes enable its use in cosmetics (e.g., skin-lightning creams), food preservation (e.g., extending shelf life of meat and fruit products), agriculture (e.g., insecticides), and pharmaceuticals (e.g., antidiabetic and anticancer agents) [1,2]. Its commercial demand, driven particularly by the global skin-lightning market, is projected to reach $38 million by 2028 [3], necessitating the development of efficient, genetically tractable, and sustainable microbial production platforms.

KA is naturally synthesized by certain *Aspergillus* and *Penicillium* species, with *Aspergillus oryzae* being the primary industrial host due to its high yield [1]. However, current production platforms face critical limitations: High-yield strains have traditionally been developed through mutagenesis, as the KA biosynthetic pathway remains incompletely elucidated, thereby constraining metabolic engineering efforts [1]. Isotope-tracing studies have shown that KA is directly synthesized from glucose via a simple pathway involving key genes *kojA* and *kojT* [[4], [5], [6], [7], [8], [9]]. While *kojA* mediates redox reactions, its precise reactants and products remain unresolved. Notably, the characterization of the KA transporter encoded by *kojT* has advanced pathway understanding, and transcriptional regulators—including KojR [10], LaeA [11], and others [[12], [13], [14], [15], [16], [17]]—have been implicated in modulating KA biosynthesis in *A. oryzae*. Among these, KojR and LaeA emerge as pivotal regulators, with genetic and transcriptomic analyses demonstrating their dual roles in activating the KA biosynthetic gene cluster.

Metabolic engineering efforts in *A. oryzae* have primarily focused on overexpressing the *koj* gene cluster (*kojA*, *kojT*, *kojR*) to enhance KA production [18]. While the engineered strain *A*. *oryzae* T58 achieved 32.5 g/L KA—a 3.2-fold improvement over wild-type—this remains substantially lower than the 96.5 g/L produced by mutagenized strain *A*. *oryzae* AR-47 [18,19]. This >66% yield gap highlights two critical needs: (i) elucidation of undefined rate-limiting steps beyond the *koj* cluster, (ii) development of alternative hosts with higher metabolic plasticity. To overcome these limitations, recent advances have expanded KA production to *Aspergillus niger* through heterologous expression of *kojA*, with deletion of the negative regulator *nrkC* yielding approximately 25 g/L in pH-controlled bioreactors [20]. Critically, this work revealed *A*. *niger*'s potential as a scalable alternative to mutagenized strains, given its industrial robustness. However, the yield gap between engineered *A*. *niger* and traditional *A*. *oryzae* mutants underscores the need for systematic pathway refinement.

**Fig. 1:**
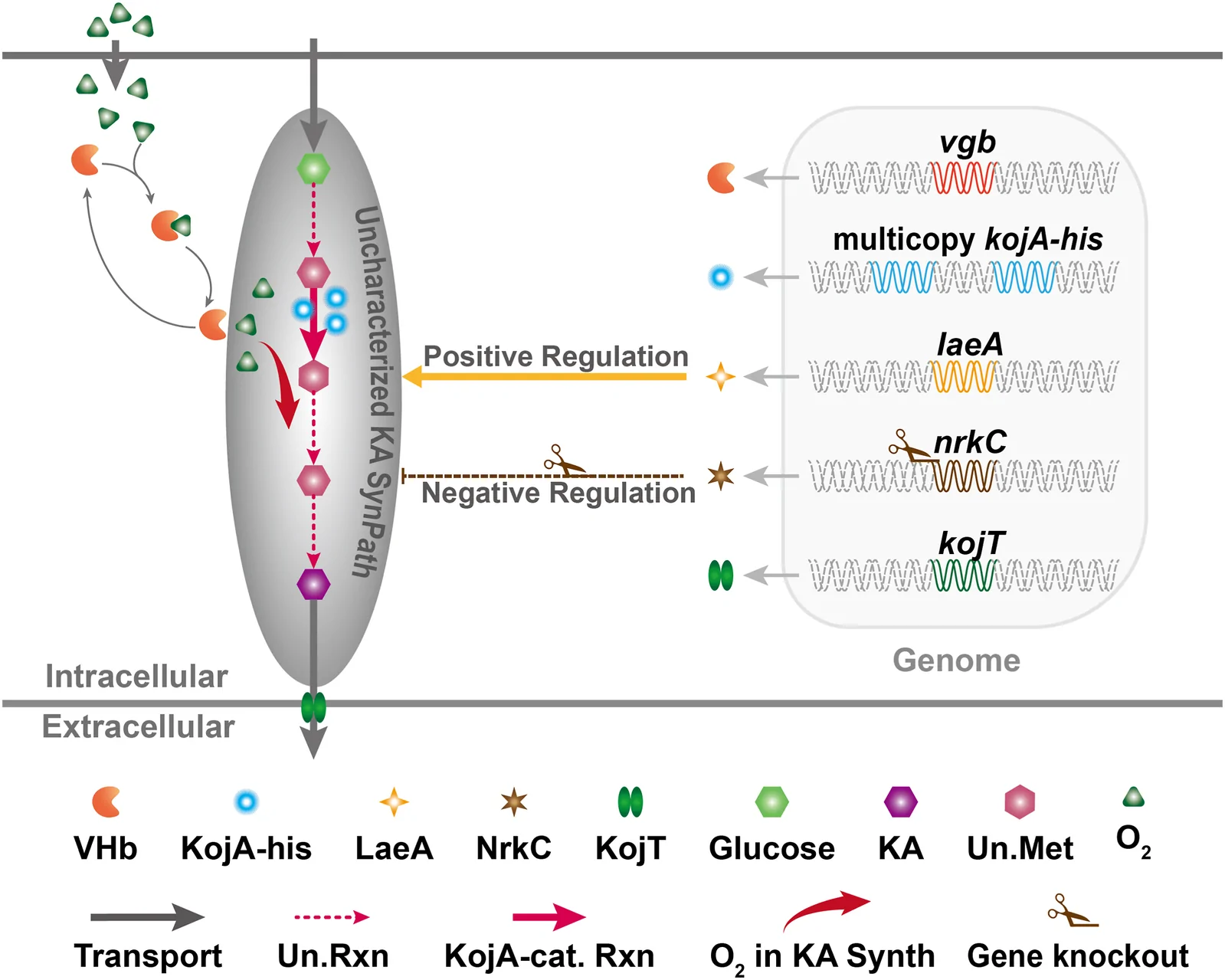
Combinatorial metabolic engineering strategy to enhance heterologous KA biosynthesis in *A*. *niger*. Abbreviations: Un.Met, uncharacterized metabolite; Un.Rxn, uncharacterized reaction; KojA-cat. Rxn, KojA-catalyzed reaction; O_2_ in KA Synth, molecular oxygen acting as the electron acceptor in kojic acid biosynthesis.

Building on these insights, we propose a combinatorial metabolic engineering strategy to enhance heterologous KA biosynthesis in *A*. *niger* (see Fig. 1). By simultaneously integrating multi-copy *kojA*-*his* integration, *kojT* overexpression, disruption of the negative regulator *nrkC*, and amplification of the global regulator *laeA* and the bacterial hemoglobin *vgb*, we address multiple metabolic bottlenecks. Coupled with tailored medium optimization, this approach demonstrates significant yield improvements. Collectively, our work demonstrates that coordinated regulation of biosynthetic flux, export efficiency, and global metabolic regulation is critical for bridging the gap between laboratory engineering and industrial-scale KA production.

## 2 Materials and methods

### 2.1 Strains and culture conditions

The *A*. *niger* strains utilized in this study are detailed in Table S1 of the Supporting Information. Strain S834, a derivative of *A*. *niger* ATCC 1015, was used as the parental strain for all subsequent genetic modifications [20]. Strain S1991 is a previously reported *A. niger* strain expressing a single-copy, untagged *kojA* gene and was used as a reference strain for comparison [20]. *Escherichia coli* JM109 was used for plasmid construction and propagation and cultured in Luria–Bertani (LB) medium at 37 °C. *Agrobacterium tumefaciens* AGL-1, used for *Agrobacterium*-mediated transformation (AMT) of *A*. *niger*, was grown at 28 °C on LB medium [21]. Spore preparation, transformant screening, and phenotypic analysis of gene overexpression or knockout mutants were carried out on potato dextrose agar (PDA), IM solid medium (IM), complete medium (CM), and minimal medium (MM), respectively, following previously described protocols [21,22]. All *A. niger* cultures were incubated at 28 °C. Where necessary, media were supplemented with 250 μg/mL hygromycin B or 100 μg/mL kanamycin for selection during strain construction and plasmid maintenance [21,22]. The KA fermentation medium used in shake flask experiments was adapted from protocols originally developed for *A. oryzae* [20]. The final composition included 100.0 g/L glucose, 2.5 g/L yeast extract, 1.0 g/L K_2_HPO_4_, 0.5 g/L MgSO_4_·7H_2_O, and 0.75 M 2-morpholinoethanesulfonic acid (MES), adjusted to pH 6.0 prior to use.

Chromogenic agar plates for *in situ* KA detection were prepared by first dissolving FeCl_3_·6H_2_O (1.0 g) and concentrated HCl (2.25 mL) in distilled water, adjusting to 100 mL to obtain the chromogenic agent; subsequently, agarose (1.5 g) was dissolved in distilled water (50 mL) at 100 °C to prepare 1.5% (w/v) agarose solution. This solution was mixed with an equal volume (50 mL) of chromogenic agent at 60 °C and immediately poured (20 mL/plate) onto KA fermentation medium plates pre-colonized by engineered strains (5–7 days at 28 °C), followed by solidification at room temperature for 10 min prior to colorimetric assessment.

### 2.2 Construction of plasmids

All plasmids utilized in this study are listed in Table S1 of the Supporting Information, and the corresponding primers are provided in Table S2. Plasmid construction was performed using the ClonExpress II One Step Cloning Kit (C112-01/02) supplied by Nanjing Vazyme Biotech Co., Ltd. (Nanjing, Jiangsu Province, China) [23]. The sequences of *kojA* (*AO090113000136*) and *AokojT* (*AO090113000138*) were amplified from the cDNA of *A*. *oryzae*, whereas the sequences of *laeA* (ASPNIDRAFT_176969) and *AngkojT* (*ASPNIDRAFT_43217*) were amplified from the cDNA of strain S834. The *vgb* overexpression plasmid (pLHVHB) [23] and the disruption plasmid (pLH1737) [20] for *AngkojT* were constructed in previous studies and are available in the laboratory.

The plasmids for expressing *kojA*-*his*, *AngkojT*, *AokojT*, and *laeA* were constructed following the same strategy as described in a previous study, using plasmid pLH454 as the backbone. For instance, the *kojA*-*his* overexpression plasmid (pKH) was generated by inserting the *kojA*-*his* coding sequence (CDS) downstream of the glyceraldehyde-3-phosphate dehydrogenase promoter (PgpdA) in pLH454 [22]. Specifically, to facilitate subsequent protein detection and purification, a 6 × His tag coding sequence was fused to the 3′ end of the *kojA* coding region, immediately upstream of the native stop codon, using a long-primer strategy. PCR amplification was performed using the cDNA of *A*. *oryzae* as the template and the primer pair KH-F/KH-R. The resulting PCR product was subsequently ligated into pLH454 digested with *Bam*HI and *Eco*RI, yielding the recombinant plasmid pKH. Following the same construction strategy, overexpression plasmids for *AngkojT*, *AokojT*, and *laeA* were generated and designated as pANKT, pAOKT, and pLAEA, respectively.

The plasmid used for *laeA* gene disruption was constructed using pLH594 [21] as the parent vector, following the same strategy described in a previous study. As an example, genomic DNA from S834 was used as the template to amplify the upstream and downstream homologous arms of *laeA* by PCR, using primer pairs laeA-UF/laeA-UR and laeA-DF/laeA-DR, respectively. The resulting fragments were sequentially ligated into the upstream and downstream regions of the hygromycin resistance cassette (*lox*P-*hph*-*lox*P) in pLH594, yielding the *AngkojT* deletion plasmid pDLAEA.

### 2.3 Construction of strains

A series of recombinant *A. niger* strains were constructed using *Agrobacterium*-mediated transformation (AMT), combined with a Cre-*lox*P system for excision of the *hph* resistance marker [23]. All plasmids were introduced into recipient strains via AMT. After transformation, *hph* marker genes flanked by *lox*P sites were removed by culturing the transformants on minimal medium (MM) plates containing doxycycline (30 μg/mL), followed by selection of hygromycin B-sensitive colonies and confirmation by PCR using primers hph-F/hph-R.

For gene overexpression, plasmids carrying target coding sequences under the control of the PgpdA were used. Transformants were selected on CM plates containing cefotaxime sodium (100 μg/mL), hygromycin B (250 μg/mL), ampicillin (100 μg/mL), and streptomycin (100 μg/mL) at 28 °C for 5 days. Colonies resistant to hygromycin B were further verified on PDA plates with 250 μg/mL hygromycin B, and correct cassette integration was confirmed by PCR.

For gene disruption, knockout cassettes containing homologous flanks and selection markers were introduced via AMT. Transformants were screened on PDA plates containing 250 μg/mL hygromycin B and MM plates supplemented with 1000 μg/mL glufosinate ammonium. Colonies showing hygromycin B resistance but glufosinate sensitivity were considered putative knockouts and confirmed by PCR.

Strain construction steps were as follows: The overexpression plasmid pKH was first introduced into *A. niger* S834 to obtain KH-R, followed by *hph* excision to generate KH-S. KH-S was subsequently retransformed with pKH twice to generate 2 KH-R/2 KH-S and 3 KH-R/3 KH-S, respectively. Using 3 KH-S as the parental strain, transformation with pANKT and pAOKT yielded AngkojT-R and AogkojT-R, respectively. The *hph* marker was then removed from AogkojT-R to generate AogkojT-S. AogkojT-S was used to construct the ΔnrkC-R mutant via transformation with pLH1497, followed by *hph* excision to obtain ΔnrkC-S. ΔnrkC-S was then transformed with pLAEA to obtain LaeA-R, from which LaeA-S was derived. LaeA-S was subsequently transformed with pLHVHB, yielding the final recombinant strain VHb-R. In parallel, the disruption strain ΔAngkojT-R was constructed by transforming pLH1737 into KH-S, and ΔLaeA-R was generated by transforming pLH1497 into ΔnrkC-S, following the same disruption and verification procedure.

### 2.4 Transcription analysis of *kojA* in different strains

The transcriptional level of the *kojA* gene in *A. niger* strains was assessed following a modified protocol based on previously reported methods [23]. After 3 days of shake-flask cultivation in KA fermentation medium, mycelia were collected and subjected to total RNA extraction. First-strand cDNA was synthesized using the PrimeScript™ II 1st Strand cDNA Synthesis Kit (TaKaRa Biomedical Technology Co., Ltd.). To examine the expression of *kojA*, quantitative real-time PCR (qRT-PCR) was performed using the SYBR Premix Taq II reagent on a StepOnePlus Real-Time PCR System (Applied Biosystems). The housekeeping gene *actin* served as the internal control. Relative expression levels were calculated using the 2^–ΔΔCt method, based on the cycle threshold (Ct) values of *kojA* and *actin* in each strain.

### 2.5 Shaking flask fermentation of *A*. *niger*

The fermentation and sampling protocol was adapted from a previously described method [23]. Engineered *A. niger* strains (1 × 10^6^ conidia/mL) were inoculated into 50 mL kA fermentation medium in 250 mL Erlenmeyer flasks and incubated at 28 °C, 200 rpm for 7 days. Culture samples were taken at selected points for KA measurement and RNA extraction. This standardized protocol was also applied across different culture media formulations during medium optimization, allowing comparative evaluation of KA production under varied nutritional conditions.

### 2.6 Bioreactor fermentation of *A*. *niger*

The fermentation protocol was adapted from previously reported methods [23]. Seed cultures were prepared by inoculating the engineered *A. niger* into 250 mL Erlenmeyer flasks containing 50 mL of the KA fermentation medium without MES, and incubated at 28 °C and 200 rpm for 24 h. The resulting seed cultures were then transferred into 1.26 L of fermentation media in 2 L bioreactors (Baoxing Biological Engineering Co. Ltd., China) and cultivated at 28 °C for 7 days. During fermentation, different medium formulations were evaluated to investigate their effects on KA production. The stirring speed was set at 250 rpm, and the aeration rate was maintained at 1 vvm (volume of air per volume of medium per minute). Fermentation broth was collected every 24 h. The supernatant was used for KA quantification, and the mycelium was harvested using pre-weighed microfiber filters and dried at 80 °C to determine dry cell weight (DCW).

### 2.7 Analytical method

KA quantification was performed using the High performance liquid chromatography (HPLC) method described in previous studies, with minor modifications [23]. HPLC was conducted using an Agilent 1260 system equipped with a variable wavelength detector. Separation was carried out on a COSMOSIL 5C18-MS-II column (4.6 mm × 250 mm), with a mobile phase consisting of methanol and 0.02 mol/L K_2_HPO_4_ in a ratio of 18:82 (v/v). The flow rate was maintained at 1.0 mL/min, the column temperature at 25 °C. Detection was performed at 273 nm.

Glucose concentration was measured using an SBA-40E biosensor analyzer (Biology Institute of Shandong Academy of Sciences, China) [23]. Sucrose and maltose concentrations were determined using HPLC (Agilent 1260 system) equipped with a refractive index detector (Agilent G1362A). The analysis was performed using a Bio-Rad Aminex HPX-87H column with 5 mM H_2_SO_4_ as the mobile phase. The column temperature was maintained at 55 °C, and the flow rate was set at 0.5 mL/min.

### 2.8 Statistical analysis

All fermentation-related data, including KA concentration, DCW, and residual sugar levels, were obtained from three biological replicates and are presented as mean ± standard deviation. Gene expression levels of *kojA* were measured from five biological replicates. Statistical analyses were performed using GraphPad Prism software. One-way or two-way analysis of variance (ANOVA) was applied as appropriate to assess statistical significance. Significance levels were defined as follows: ∗P < 0.05, ∗∗P < 0.01, ∗∗∗P < 0.001 and ∗∗∗∗P < 0.0001.

## 3 Results and discussion

### 3.1 Copy number optimization of *kojA* enhances KA production

Previous research has characterized the KA biosynthetic pathway as a concise three-step route originating from glucose [2,20]. While the specific biochemical roles of each enzymatic step remained incompletely defined, the *kojA* gene—encoding the oxidoreductase KojA—has been identified as essential for KA formation [2,20]. In heterologous production systems using *A*. *niger*, which does not naturally synthesize KA, introduction and overexpression of *kojA* alone was sufficient to enable KA biosynthesis, highlighting its pivotal function [20]. This observation led to the hypothesis that increasing *kojA* expression may enhance KA production. To investigate this possibility, the strong constitutive *gpdA* promoter was utilized to drive *kojA* transcription, and expression was further elevated by increasing the gene copy number. A C-terminal 6 × His tag (KojA-His) was appended to each copy to enable simplified downstream purification.

**Fig. 2:**
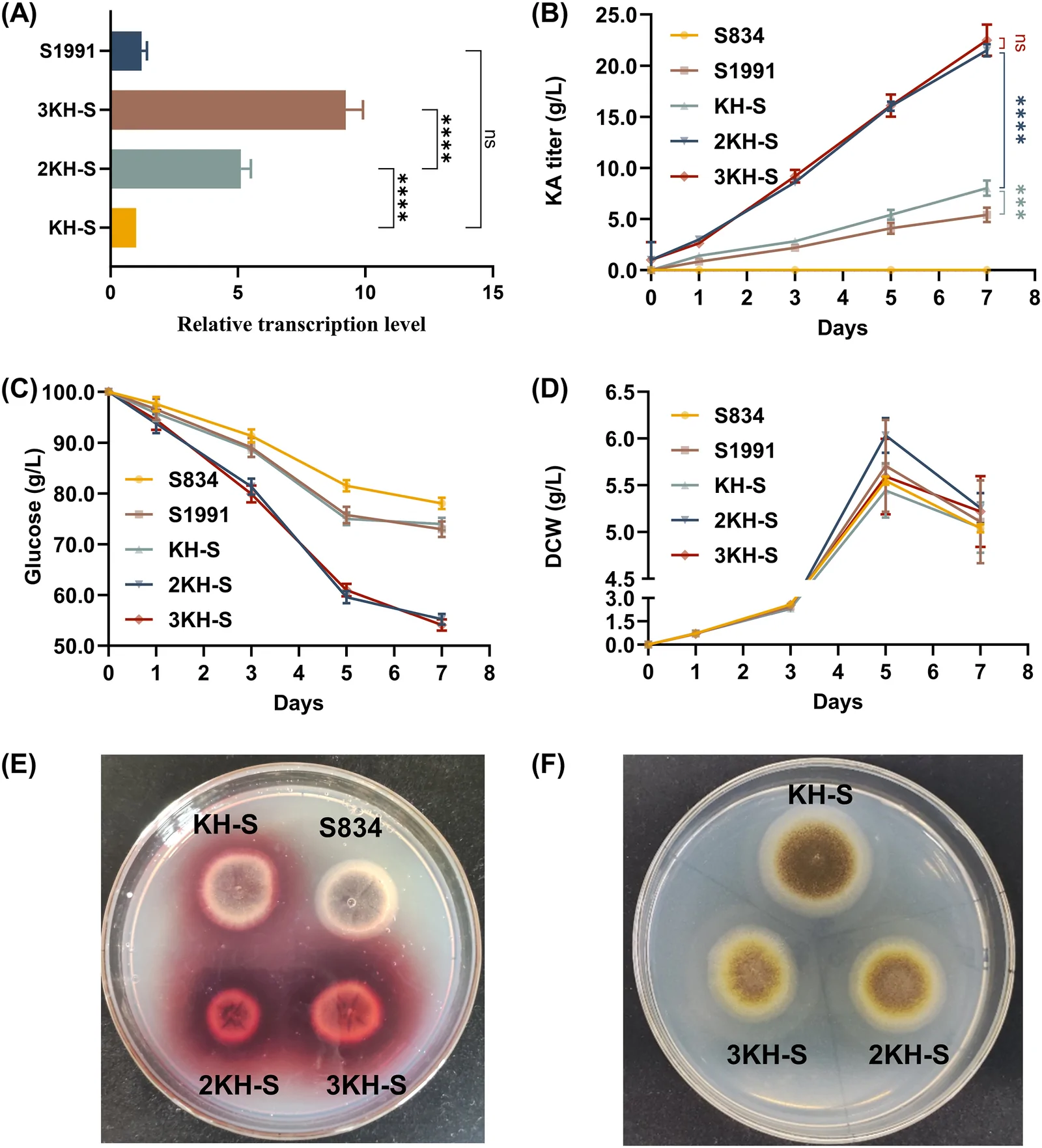
Effect of *kojA*-*his* gene copy number on KA production in engineered strains. S834 was the parental strain. S1991 (single-copy *kojA* without His-tag, constructed previously) was used as a reference strain. (A) Relative transcription levels of *kojA*-*his* in engineered strains KH-S (single copy), 2 KH-S (double copy), and 3 KH-S (triple copy), compared to S1991 on day 3 of shake-flask fermentation, determined by qRT-PCR. (B) KA titer, (C) Residual glucose and (D) Dry cell weight during the 7-day fermentation period. (E) Qualitative colorimetric detection of KA production on chromogenic agar plates, showing purple pigment formation in engineered strains compared with parental strain S834. (F) Conidiation phenotypes on PDA plates. Data represent mean ± SD (n = 3 biological replicates). Statistical significance was determined using one-way ANOVA (A) or two-way ANOVA (B–D) (∗p < 0.05, ∗∗p < 0.01, ∗∗∗p < 0.001, ∗∗∗∗p < 0.0001).

Using a constitutive promoter PgpdA, expression cassettes of *kojA*-*his* were integrated in one, two, or three copies into the genome of *A*. *niger* strain S834, a background strain engineered to eliminate citric and oxalic acid by-product formation. For each integration, transformants were initially selected based on hygromycin resistance (*hph*), yielding intermediate strains KH-R, 2 KH-R, and 3 KH-R. The *hph* marker was subsequently excised through marker recycling to generate the final marker-free, hygromycin-sensitive strains KH-S (1 × *kojA*-*his*), 2 KH-S (2 × ), and 3 KH-S (3 × ), respectively. Quantitative RT-PCR performed on day 3 of shake-flask fermentation demonstrated that *kojA*-*his* transcript levels in 2 KH-S and 3 KH-S increased approximately 5.1-fold and 9.2-fold, respectively, relative to KH-S (Fig. 2A). These results confirm both successful transcription and a positive correlation between gene copy number and mRNA abundance. On KA fermentation agar, purple pigmentation characteristic of KA production appeared exclusively in the engineered strains by day 3, with color intensity increasing from KH-S to 2 KH-S and 3 KH-S. This gradient matched the gene copy number, supporting a dose-dependent increase in KA accumulation (Fig. 2E).

In 7-day fermentations initiated with 100 g/L glucose, KA production steadily increased in all engineered strains, with final titers of 21.5 g/L in 2 KH-S and 22.5 g/L in 3 KH-S, compared to 8.0 g/L in KH-S and negligible levels in the parental strain S834 (Fig. 2B). Glucose consumption followed a similar trend. residual glucose levels on day 7 were 74.0 g/L (KH-S), 55.2 g/L (2 KH-S), and 54.1 g/L (3 KH-S) (Fig. 2C). Yield calculations based on glucose consumption revealed 0.48 g/g (2 KH-S) and 0.49 g/g (3 KH-S), substantially exceeding the 0.31 g/g observed in KH-S. DCW peaked between 5 and 6 g/L on day 5 across all strains, followed by a modest decline (Fig. 2D). No significant differences in biomass were detected among the engineered strains, indicating that increasing *kojA*-*his* copy number did not impair growth or cellular physiology. KA accumulation continued during the stationary phase, suggesting that biosynthesis remained active beyond peak biomass.

Comparison with a previously reported single-copy strain, S1991 [20], which expresses untagged KojA, revealed that KH-S (tagged KojA-His) produced 48.8% more KA (8.0 g/L vs. 5.4 g/L) (Fig. 2A). However, qRT-PCR analysis indicated no significant difference in *kojA* transcript levels between the two strains (Fig. 2A). This observation indicates that the presence of the His tag is associated with increased KA production, but does not allow direct conclusions regarding the underlying mechanism. Previous studies have reported that affinity tags, including His tags, may influence enzyme properties such as stability or activity [[24], [25], [26]]. Accordingly, the enhanced KA yield observed in KH-S could be related to altered biochemical characteristics of KojA-His. However, in the absence of protein-level or enzymatic activity data, the specific mechanism responsible for this effect remains unresolved.

Although the *kojA-his* transcript level in 3 KH-S was ∼1.8-fold higher than in 2 KH-S, no further increase in KA titer or yield was observed under shake-flask conditions. Both strains achieved similar final titers (∼22 g/L) and glucose-to-KA conversion ratios (∼0.62–0.63 g/g), indicating a saturation point beyond which additional gene copies conferred limited benefit. However, 3 KH-S maintained a slight advantage in final KA output, making it a suitable host for further metabolic engineering. Notably, a gradual decrease in pigmentation and conidiation from KH-S to 3 KH-S was observed on PDA plates (Fig. 2F), suggesting that excessive gene dosage or insertional effects may impact developmental traits.

The study identified three distinct phases in the dosage effects of *kojA*: First, a linear phase (1 to 2 gene copies) resulted in a 2.7-fold increase in KA titer; second, a plateau phase (2 to 3 copies) yielded less than 5% improvement; and third, a physiological trade-off phase, where the 3-copy strain (3 KH-S) exhibited reduced conidiation. Although 3 KH-S showed marginal titer advantage, its 9.2-fold transcriptional activation and stable genomic integration provided a robust platform for subsequent transporter engineering.

### 3.2 Enhanced KA efflux by overexpression of the KojT transporter

To address downstream bottlenecks in KA biosynthesis, the secretion step was evaluated as a potential limiting factor. In microbial systems, insufficient export of secondary metabolites is widely recognized to constrain overall production efficiency by imposing product-associated metabolic and regulatory limitations, even when the biosynthetic capacity is enhanced. Therefore, enhancing KA efflux capacity was explored as a complementary strategy to the increased biosynthetic flux achieved by *kojA* amplification.

To further assess whether kojic acid (KA) may impose physiological constraints at high concentrations, we performed an additional growth inhibition assay using exogenously supplied KA. When *A. niger* S834 and *A. oryzae* NRRL3488 were cultivated on PDA plates for 5 days with increasing KA concentrations (0–50 g/L), KA exhibited a clear concentration-dependent inhibitory effect. Colony size gradually decreased with increasing KA concentration; marked growth inhibition was observed at 40 g/L, and no visible colony growth occurred at 50 g/L (Fig. S1). Under identical conditions, both strains showed highly similar inhibition patterns, indicating comparable tolerance to KA. These results demonstrate that sufficiently high KA concentrations can inhibit *A. niger* growth, supporting the rationale for investigating KA export capacity in production systems.

**Fig. 3:**
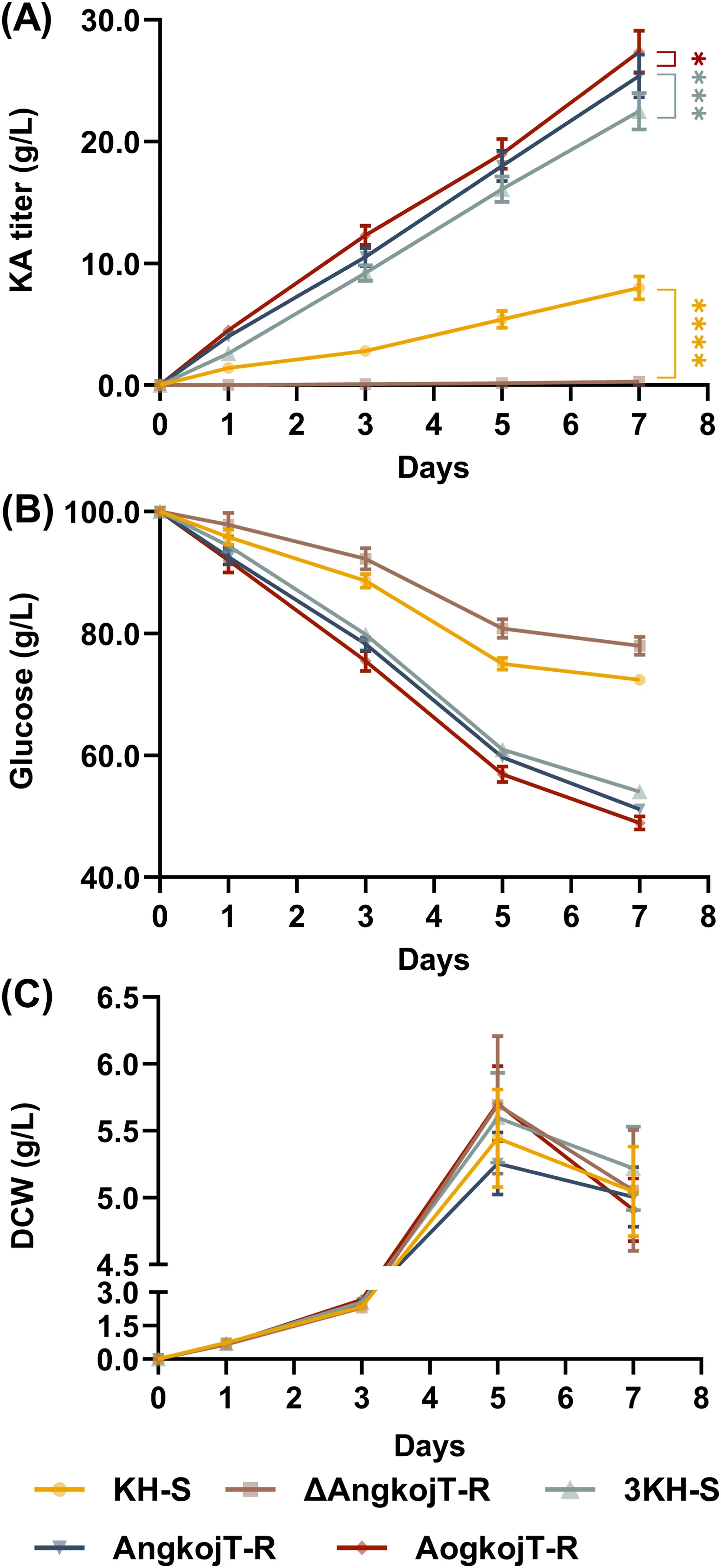
Functional validation and enhancement of KA export through transporter engineering in *A. niger*. AngkojT was deleted in the KH-S (1 × *kojA-his*) background to generate ΔAngkojT-R, and AngkojT or AokojT was overexpressed in the high-producing 3 KH-S (3 × *kojA-his*) background to generate AngkojT-R and AokojT-R, respectively. (A) KA titer, (B) residual glucose concentration, and (C) dry cell weight (DCW) after 7-day shake-flask fermentation. Data are mean ± SD (n = 3). Two-way ANOVA. (∗p < 0.05, ∗∗p < 0.01, ∗∗∗p < 0.001, ∗∗∗∗p < 0.0001).

Functional conservation of KA transporters was first assessed based on sequence similarity. AngKojT (ASPNIDRAFT_43217, GenBank EHA28474.1), the predicted endogenous KA exporter in *A*. *niger*, shares 78% amino acid identity with AoKojT from *A*. *oryzae* (AO090113000138), suggesting conserved functionality across species. To validate the functional relevance of *AngkojT*, the gene was deleted in a 1 × *kojA-his* background (KH-S), generating strain ΔAngkojT-R. After 7 days of shake-flask fermentation, the strain ΔAngkojT-R accumulated only 0.3 g/L KA, in stark contrast to 8.0 g/L in KH-S (Fig. 3A). Residual glucose remained high at 78.0 g/L in ΔAngkojT-R compared to 72.4 g/L in KH-S (Fig. 3B), and biomass accumulation was comparable (5.1 g/L dry weight in both strains; Fig. 3C). Collectively, these results indicate that inactivation of *AngkojT* severely disrupts KA accumulation in the culture broth, confirming that *AngkojT* is essential for efficient KA export under the tested fermentation conditions.

Having established the functional importance of KojT-mediated export, we next tested whether enhancing efflux capacity could further improve productivity in a high-flux background. *AngkojT* and *AokojT* were overexpressed in the high-producing 3 KH-S background (3 × *kojA*-*his*, marker-free), generating the engineered strains AngkojT-R and AokojT-R, respectively. After 7 days of fermentation, KA titers increased from 22.5 g/L in 3 KH-S to 25.4 g/L in AngkojT-R (an increase of 12.9%) and 27.4 g/L in AokojT-R (a 21.8% increase) (Fig. 3A). Correspondingly, residual glucose concentrations decreased from 54.1 g/L in 3 KH-S to 51.1 g/L in AngkojT-R and 48.9 g/L in AokojT-R (Fig. 3B), indicating more efficient substrate utilization. Biomass levels remained comparable across the three strains, ranging from 4.9 to 5.2 g/L (Fig. 3C), indicating no major physiological burden from transporter overexpression. In terms of carbon conversion efficiency, the KA yield (g/g glucose consumed) improved from 0.49 in 3 KH-S to 0.52 in AngkojT-R (an improvement of 6.1%) and 0.54 in AokojT-R (an improvement of 9.4%). These gains in both titer and yield highlight that KA efflux represents a critical rate-limiting step in the production pathway, particularly under conditions of elevated biosynthetic flux. Notably, the heterologous transporter AoKojT outperformed the native AngKojT, and the AoKojT-R strain was therefore selected as the optimal production host for subsequent optimization.

Overall, these findings indicate that KA production is jointly governed by biosynthetic capacity (Section 3.1) and export efficiency (this section). Even with enhanced pathway flux, export capacity can remain limiting, and relieving this constraint improves both productivity and carbon conversion. Beyond pathway capacity and export, additional layers of global regulation may further influence KA biosynthesis. Therefore, we next investigated two complementary approaches to modulate regulatory control: derepression of negative regulation via *nrkC* deletion and enhancement of positive regulation through *laeA* and *vgb* overexpression.

### 3.3 Enhancing KA production via global regulatory strategies

Transporter engineering described previously confirmed that KA export is a rate-limiting step and yielded the high-producing strain AokojT-R. On this basis, further improvements were sought by targeting global regulatory mechanisms. Because the KA biosynthetic pathway remains largely unresolved—with only *kojA* and the transporter *kojT* clearly characterized—other reactions and genes involved can be regarded as a “black box” [1,2]. Therefore, manipulating upstream regulators and oxygen supply was considered a rational strategy to further unlock this pathway and enhance KA production.

**Fig. 4:**
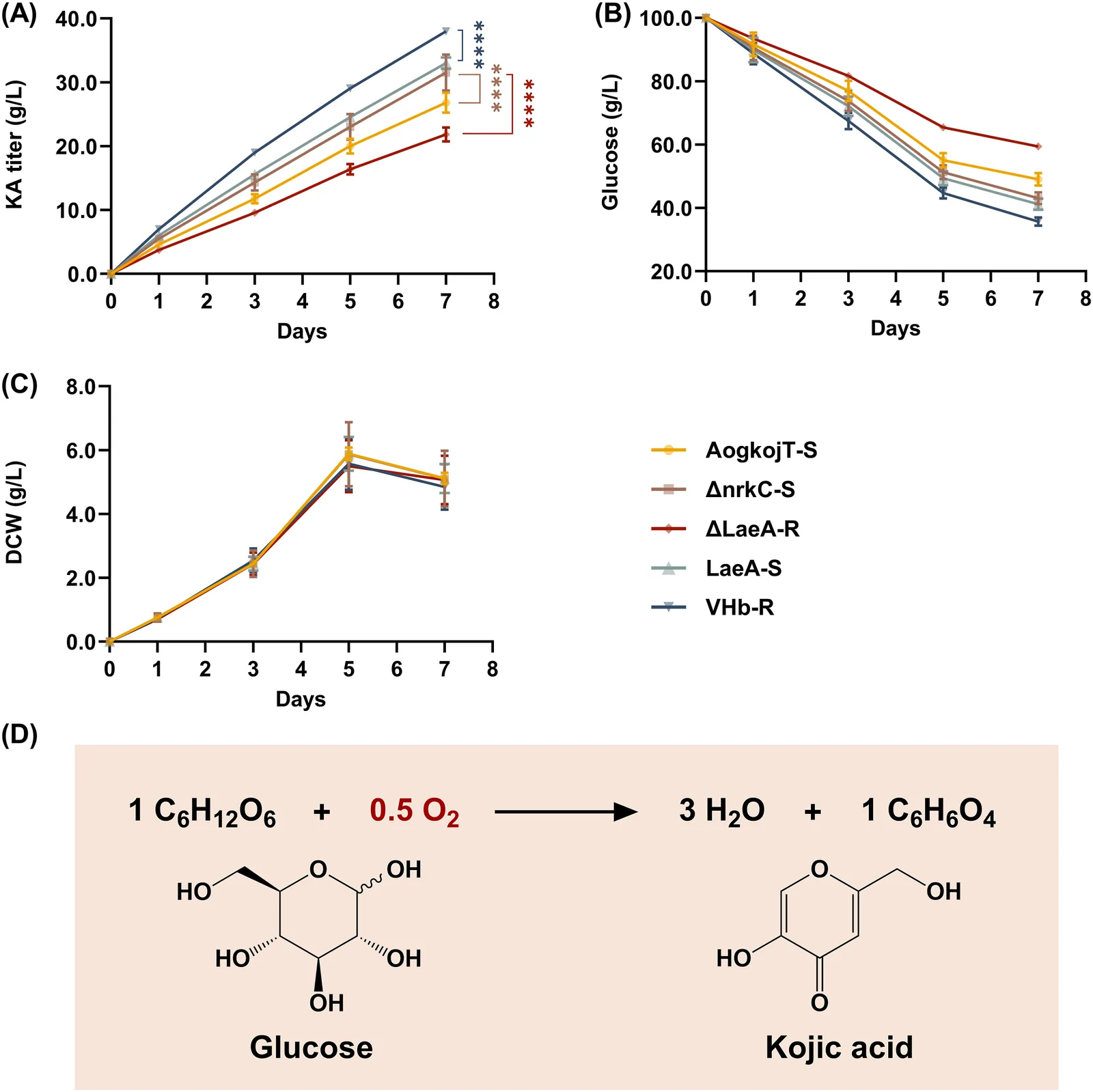
Sequential regulatory and oxygen-supply engineering further enhances KA production in *A. niger.* AogkojT-S (3 × *kojA-his* with *AokojT* overexpression) served as the parental strain. Deletion of *nrkC* generated ΔnrkC-S; subsequent *laeA* deletion and overexpression yielded ΔLaeA-R and LaeA-S, respectively; expression of VHb in the LaeA-S background generated VHb-R. (A) KA titer, (B) residual glucose concentration, and (C) dry cell weight (DCW) after 7-day shake-flask fermentation. (D) Stoichiometric relationship between glucose consumption and KA formation, indicating oxygen involvement. Data are mean ± SD (n = 3). Two-way ANOVA. (∗p < 0.05, ∗∗p < 0.01, ∗∗∗p < 0.001, ∗∗∗∗p < 0.0001).

Recent studies in the KA-producing *A*. *niger* S2132 strain identified four genes, *nrkA*, *nrkB*, *nrkC*, and *nrkD*, that negatively regulate KA biosynthesis [20]. Individual deletion of any of these genes markedly increased KA titers, while simultaneous deletion of all four did not lead to further improvement, suggesting their involvement in a common regulatory process. Among them, *nrkC* deletion had the most pronounced effect. Based on these findings, the selection marker in AokojT-R was first excised to generate the marker-free strain AokojT-S, which served as the host for targeted *nrkC* deletion. This yielded the strain ΔnrkC-R, from which the marker *hph* was subsequently removed to obtain the final strain ΔnrkC-S. In shake-flask fermentation, KA titer increased from 26.8 g/L in AokojT-S to 31.5 g/L in ΔnrkC-S, with residual glucose decreasing from 49.0 g/L to 43.0 g/L (Fig. 4A and B). The yield improved from 0.53 g/g to 0.55 g/g, while DCW remained essentially unchanged at approximately 5.1 g/L (Fig. 4C), indicating that *nrkC* deletion effectively relieved negative regulation without impairing growth.

While *nrkC* deletion removed production inhibition, we next sought to actively enhance biosynthetic flux through the global regulator LaeA. LaeA is known in filamentous fungi to interact with members of the Velvet family (VeA, VelB, VelC, VelD, and others) to form regulatory complexes that fine-tune secondary metabolite biosynthesis [27]. It has been reported that the disruption of LaeA in *A*. *oryzae* drastically reduces or abolishes KA production, whereas gene complementation restores it, highlighting LaeA as a key positive regulator of KA biosynthesis [11]. Importantly, although *kojA* is an essential gene for KA biosynthesis, it is unlikely to represent the sole enzymatic step involved in the overall metabolic process, and additional unknown or auxiliary reactions may also contribute to KA formation. On this basis, *laeA* was incorporated not solely to modulate *kojA* expression, but to assess its potential regulatory role on other LaeA-responsive components of the KA metabolic network.

To assess whether LaeA exerts similar regulatory effects in *A*. *niger*, *laeA* was first knocked out in the ΔnrkC-S background, generating the strain ΔLaeA-R. Fermentation results showed a substantial decrease in KA production to 21.8 g/L, representing a 30.6% reduction compared to the parental strain ΔnrkC-S (31.5 g/L), while DCW remained essentially unchanged (Fig. 4A and C). This result confirmed that *laeA* also plays a positive role in promoting KA biosynthesis in *A*. *niger*, even under heterologous pathway conditions. Subsequently, *LaeA* was overexpressed in the ΔnrkC-S background, resulting in the strain LaeA-R. After marker removal, the final LaeA overexpression strain, LaeA-S, was obtained. Compared to ΔnrkC-S, LaeA-S exhibited a modest improvement in KA production, reaching 33.0 g/L (Fig. 4A). Residual glucose decreased to 41.2 g/L, and the yield increased to 0.56 g/g (Fig. 4B). Biomass remained consistent (Fig. 4C), suggesting that *LaeA* overexpression primarily enhanced secondary metabolism without significantly affecting growth. However, the limited improvement compared to the parental ΔnrkC-S strain suggests that although LaeA is necessary for enabling KA production, its abundance is not rate-limiting under the current engineering configuration. One plausible explanation for this observation is that *kojA*, the core gene responsible for KA biosynthesis, is driven by the strong constitutive P*gpdA* promoter in the heterologous system, which likely bypasses direct LaeA-dependent regulation. As a consequence, the regulatory impact of LaeA overexpression may be diluted in systems employing strong heterologous promoters, representing an inherent limitation of this engineering strategy. Nevertheless, LaeA may still play an essential role in maintaining the broader metabolic and regulatory network required for KA production, potentially through the regulation of auxiliary or currently unidentified reactions that support the biosynthetic process. While these steps appear to be indispensable, they do not constitute the major rate-limiting factors, which explains why *laeA* deletion results in a sharp decline in KA production, whereas laeA overexpression yields only marginal improvements in titer. Taken together, these findings suggest that *laeA* functions as an enabling but non-dominant regulatory element in the present system.

In addition to genetic regulation, oxygen availability is another critical factor. The stoichiometry of the reaction converting glucose to KA (Fig. 4D) indicates that oxygen is involved in the transformation process, with each molecule of glucose requiring 0.5 molecules of oxygen. This suggests that limited oxygen supply may constrain KA production. Bacterial hemoglobin (VHb), encoded by the *vgb* gene from *Vitreoscilla*, is widely employed to enhance oxygen transfer and utilization in various microorganisms, including filamentous fungi [23]. VHb binds oxygen efficiently even under microaerobic conditions and delivers it to terminal oxidases, thereby improving respiratory efficiency and overall metabolism. Overexpression of VHb in the LaeA-S background produced strain VHb-R, which achieved the highest KA titer of 38.0 g/L (Fig. 4A) and the highest yield of 0.59 g/g, with residual glucose reduced to 35.7 g/L (Fig. 4B). Although dry cell weight showed a slight decrease to 4.85 g/L (Fig. 4C), overall growth remained satisfactory, indicating that improved oxygen supply effectively unlocked further biosynthetic potential.

Among the successive modifications, *nrkC* deletion yielded the most significant improvement in KA production, with a 17.5% increase over the parental strain AokojT-S. Additional LaeA overexpression and VHb expression provided incremental gains of 4.8% and 15.2%, respectively, demonstrating the cumulative but diminishing returns of global regulation and oxygen enhancement strategies. The differential impact of *laeA* deletion and overexpression further suggests that while LaeA-responsive pathways support KA biosynthesis in *A. niger*, they may not constitute the primary bottlenecks. Future efforts may benefit from mapping these downstream pathways in greater detail and identifying additional control points within this “black box” to more precisely direct metabolic flux toward KA.

### 3.4 Medium optimization and bioreactor validation

In this study, a basal fermentation medium comprising glucose (100 g/L) and yeast extract (2.5 g/L) was employed, supplemented with 0.75 M MES buffer to stabilize the extracellular pH at 6.0 during preliminary fermentation assays [20]. The use of buffering agents is common in *A. niger* cultivation, as both fungal growth and secondary metabolite biosynthesis in this species are highly sensitive to extracellular pH fluctuations [28]. Owing to its pKa value and effective buffering range (pH 5.5 - 6.7), MES is particularly suitable for maintaining mildly acidic to near-neutral pH conditions and is therefore frequently applied during screening-stage fermentations to improve experimental reproducibility.

MES does not directly participate in core cellular metabolic pathways and has long been classified as a biologically compatible Good's zwitterionic buffer [29]. However, accumulating evidence suggests that such buffers cannot be assumed to be completely physiologically inert. Studies on related Good's buffers, including HEPES, TES, and BES, have demonstrated that these compounds may be taken up by cells or influence membrane-associated processes in a context- and concentration-dependent manner, potentially affecting transport activities, ion gradients, or other physiological properties [30]. In addition, Good's buffers have been reported to interact with metal ions and metal–protein systems at commonly used concentrations, indicating that external buffering agents may introduce variables beyond pH regulation alone [31]. Although these effects are often subtle and system-specific, they highlight the importance of carefully evaluating the role of exogenous buffers in metabolic engineering studies.

While the MES-buffered medium successfully supported baseline cell growth and KA biosynthesis, the inclusion of MES increased operational complexity and cost, and potentially introduced additional experimental variables unrelated to intrinsic cellular metabolism. These considerations prompted us to reassess whether stable pH control—and consequently high KA production—could be achieved through intrinsic medium design rather than reliance on exogenous buffering. Therefore, the objective of this section was to optimize the fermentation medium for enhanced KA production and to investigate whether pH stability within a favorable range could be maintained through rational adjustment of medium composition alone, ultimately eliminating the need for MES buffer and enabling a simpler, more scalable cultivation process.

**Fig. 5:**
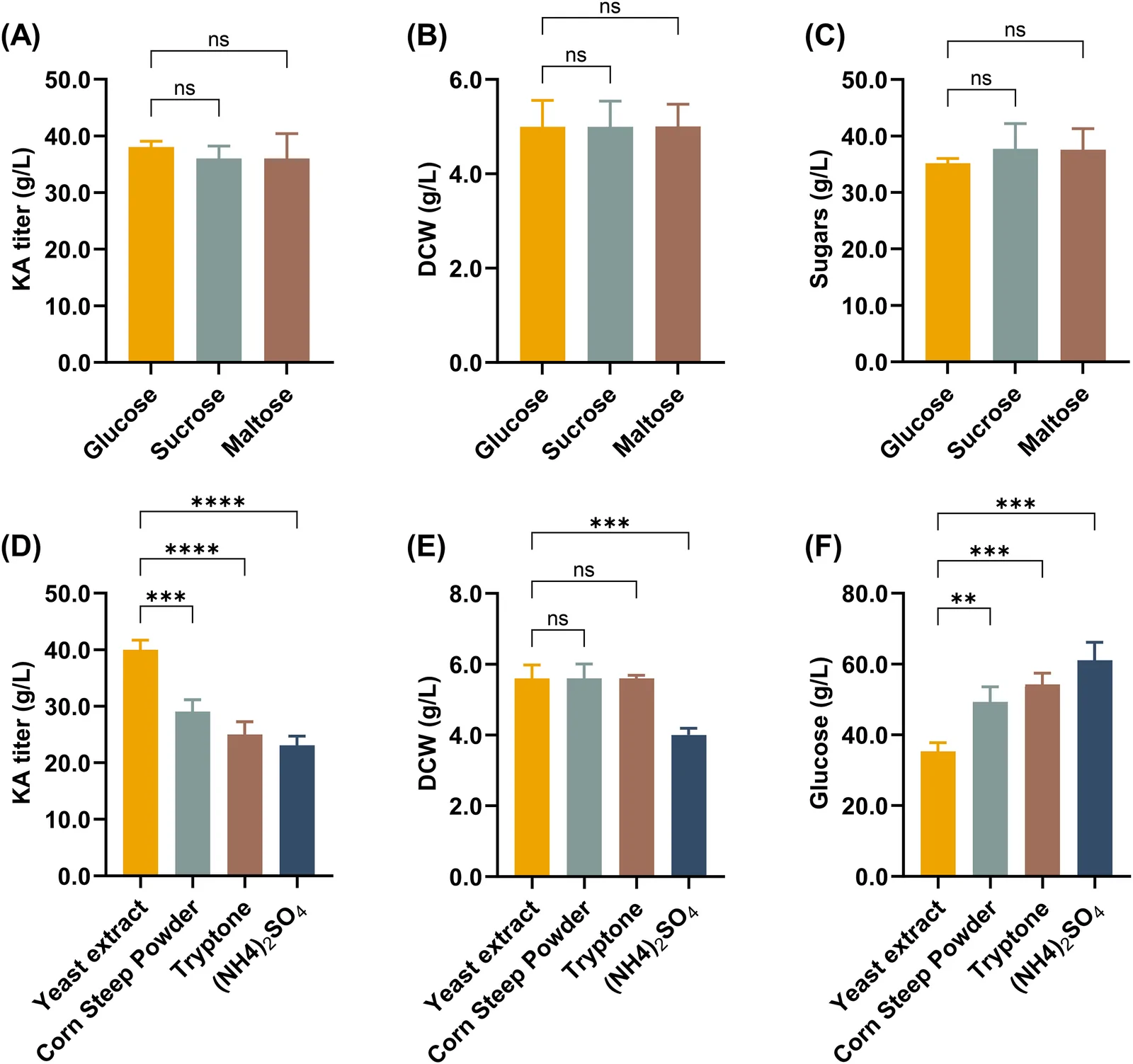
Effects of carbon and nitrogen sources on KA production by the engineered *A. niger* strain VHb-R. (A–C) KA titer, dry cell weight (DCW), and residual glucose concentration after 7-day shake-flask fermentation with different carbon sources. (D–F) KA titer, DCW, and residual glucose concentration after 7-day fermentation with different nitrogen sources. Data are mean ± SD (n = 3). Two-way ANOVA. (∗p < 0.05, ∗∗p < 0.01, ∗∗∗p < 0.001, ∗∗∗∗p < 0.0001).

To provide a reliable basis for medium optimization, a thorough review of prior studies on KA fermentation by various *Aspergillus* species was conducted, summarizing key findings in Table S3 of Supporting Information, which compiles data from 37 relevant studies. The reviewed strains included *A. oryzae*, *A. flavus*, *A. candidus*, *A. sojae*, and others. These studies predominantly employed glucose (60–200 g/L) and sucrose (70–220 g/L) as carbon sources, with glucose being the most widely used substrate. Occasionally, dextrin was also utilized. Based on this literature, the effect of different carbon sources—glucose, sucrose, and maltose—on KA production by the engineered strain VHb-R was initially assessed in 7-day shake flask fermentations. When cultivated with glucose, sucrose, or maltose as the sole carbon source, VHb-R produced KA titers of 38.0, 36.0, and 37.3 g/L, respectively (Fig. 5A). Biomass accumulation was comparable across all groups (∼5.0 g/L), while residual sugar concentrations ranged from 35.2 to 38.1 g/L (Fig. 5B and C). The KA yields exhibited comparable values, ranging from 0.57 to 0.59 g kA/g sugar. One-way ANOVA revealed no statistically significant differences in KA production, biomass, or residual sugar among the three carbon sources. These results suggest that the type of carbon source had minimal differential effects on KA biosynthesis, likely due to the high metabolic flexibility of *A. niger*. Nevertheless, glucose was selected as the carbon source for subsequent optimization, based on its slightly higher titer and yield, as well as its metabolic simplicity and broad industrial availability.

The literature review (Table S3) further revealed that nitrogen source selection plays a crucial role in KA production across *Aspergillus* species. Yeast extract (1–20 g/L) was identified as the most effective organic nitrogen source, with an optimal concentration range of 5–10 g/L. Peptone (4–7 g/L) showed moderate performance, while inorganic salts (e.g., NH_4_NO_3_ at 1.13–5 g/L) generally resulted in lower KA titers. With glucose established as the carbon source, the impact of different nitrogen sources on KA production was evaluated using yeast extract, peptone, corn steep powder, and ammonium sulfate (each at 5 g/L). The strain VHb-R utilizing yeast extract as the nitrogen source achieved the highest KA titer (40.0 g/L), significantly exceeding the titers observed with peptone (25.0 g/L), corn steep powder (29.0 g/L), and ammonium sulfate (23.0 g/L) (Fig. 5D). Moreover, the yeast extract group exhibited the highest yield (0.62 g kA/g glucose), compared to 0.55–0.59 g kA/g glucose in the other groups. Biomass in the yeast extract group reached 5.6 g/L, which was comparable to that of peptone and corn steep powder but notably higher than the 4.0 g/L recorded with ammonium sulfate (Fig. 5E). Residual sugar levels further supported the superior performance of yeast extract, with the lowest residual sugar concentration (35.3 g/L), which positively correlated with both KA titer and yield (Fig. 5F). These findings are consistent with the literature, where yeast extract is the most commonly used organic nitrogen source for KA fermentation, with an optimal concentration range that supports high yields in many *Aspergillus* strains. While industrial-scale processes may benefit from alternative nitrogen supplementation strategies (e.g., wheat germ supplementation) [32], yeast extract remains the preferred choice for laboratory and small-scale fermentation.

**Fig. 6:**
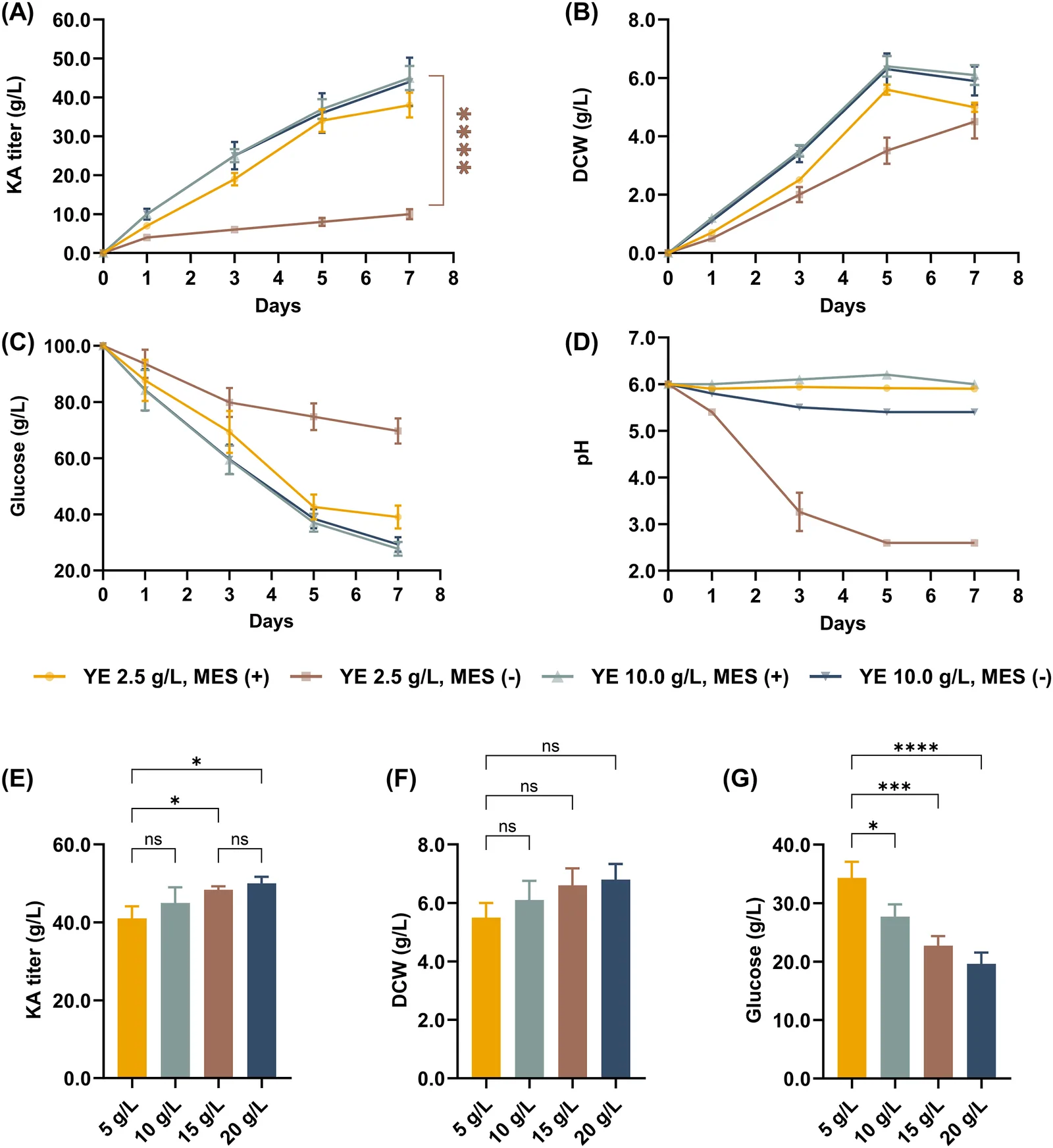
Effects of yeast extract (YE) concentration and buffering conditions on KA production by *A. niger* strain VHb-R. (A–D) KA titer, dry cell weight (DCW), residual glucose concentration, and pH during 7-day shake-flask fermentation under four conditions: YE 2.5 g/L, MES (+); YE 2.5 g/L, MES (−); YE 10.0 g/L, MES (+); and YE 10.0 g/L, MES (−). MES (+) indicates supplementation with 0.75 M MES buffer, whereas MES (−) indicates no MES addition. (E–G) KA titer, DCW, and residual glucose concentration under increasing YE concentrations (5–20 g/L) in MES-free medium. Data are mean ± SD (n = 3). Two-way ANOVA. (∗p < 0.05, ∗∗p < 0.01, ∗∗∗p < 0.001, ∗∗∗∗p < 0.0001).

Subsequently, the effect of yeast extract concentration—both with and without MES buffer—on KA production and pH regulation was investigated. Under low nitrogen conditions (2.5 g/L yeast extract), the MES-buffered group achieved a KA titer of 38.0 g/L, whereas the non-buffered group produced only 10.0 g/L (Fig. 6A). pH monitoring revealed a rapid decline to 2.8 by day 3 and further to 2.5 by the end of fermentation in the absence of MES, while the pH in the buffered group remained stable around 6.0 (Fig. 6D). These observations suggest that excessive acidification may indirectly limit KA production, highlighting the importance of maintaining pH within a favorable range. Under optimized nitrogen conditions, stable pH control could be achieved without MES supplementation, indicating that pH stability rather than the presence of MES itself is the key factor influencing KA biosynthesis in this system. Under high nitrogen conditions (10 g/L yeast extract), KA production reached 44.0–45.0 g/L in both buffered and non-buffered groups (Fig. 6A). Notably, in the absence of MES, the pH remained relatively stable at approximately 5.5 (Fig. 6D), indicating that higher nitrogen concentrations may contribute to pH stabilization, potentially mitigating the need for external buffering. According to the literature, optimal pH values for KA production vary significantly among *Aspergillus* species, demonstrating strong strain-specific preferences. For example, acidic conditions (pH 3.0–4.5) are most favorable for *A. oryzae* and *A. flavus*, with a peak production of 139.24 g/L observed for *A. oryzae* Ao-4 at pH 3.0 (Table S3). In contrast, neutral pH (6.0–6.5) is suitable only for a limited number of strains such as *A. candidus*, while extreme acidity (pH 2.1) has only been reported for certain mutants of *A. terreus* (Table S3). In comparison, the present study indicates that *A. niger* performs optimally at relatively higher pH values (above 5.4), emphasizing the importance of tailoring pH regulation to the specific strain.

To determine the optimal nitrogen level, additional experiments were conducted with yeast extract concentrations ranging from 5 to 20 g/L (without MES buffering). KA production increased progressively with higher nitrogen input, rising from 41.0 g/L at 5 g/L yeast extract to 50.0 g/L at 20 g/L (Fig. 6E). Although no significant difference in KA titer was observed between adjacent concentrations (i.e., 5 vs. 10 g/L and 15 vs. 20 g/L), both the 15 and 20 g/L groups exhibited significantly higher titers than the 5 g/L group (Fig. 6D). Biomass increased accordingly, from 5.5 to 6.8 g/L, and showed a strong positive correlation with KA titer (Fig. 6F). Residual sugar concentrations decreased from 35.3 to 18.4 g/L (Fig. 6G), suggesting enhanced substrate utilization with increasing nitrogen availability. Notably, the KA yields remained stable (0.61∼0.63 g kA/g glucose) across all conditions. Meanwhile, pH levels in all groups remained above 5.4 throughout fermentation. Given that 15 g/L yeast extract supported near-maximal KA production in shake flask experiments, this concentration, along with 10 and 5 g/L, was selected for further evaluation in bioreactor studies.

**Fig. 7:**
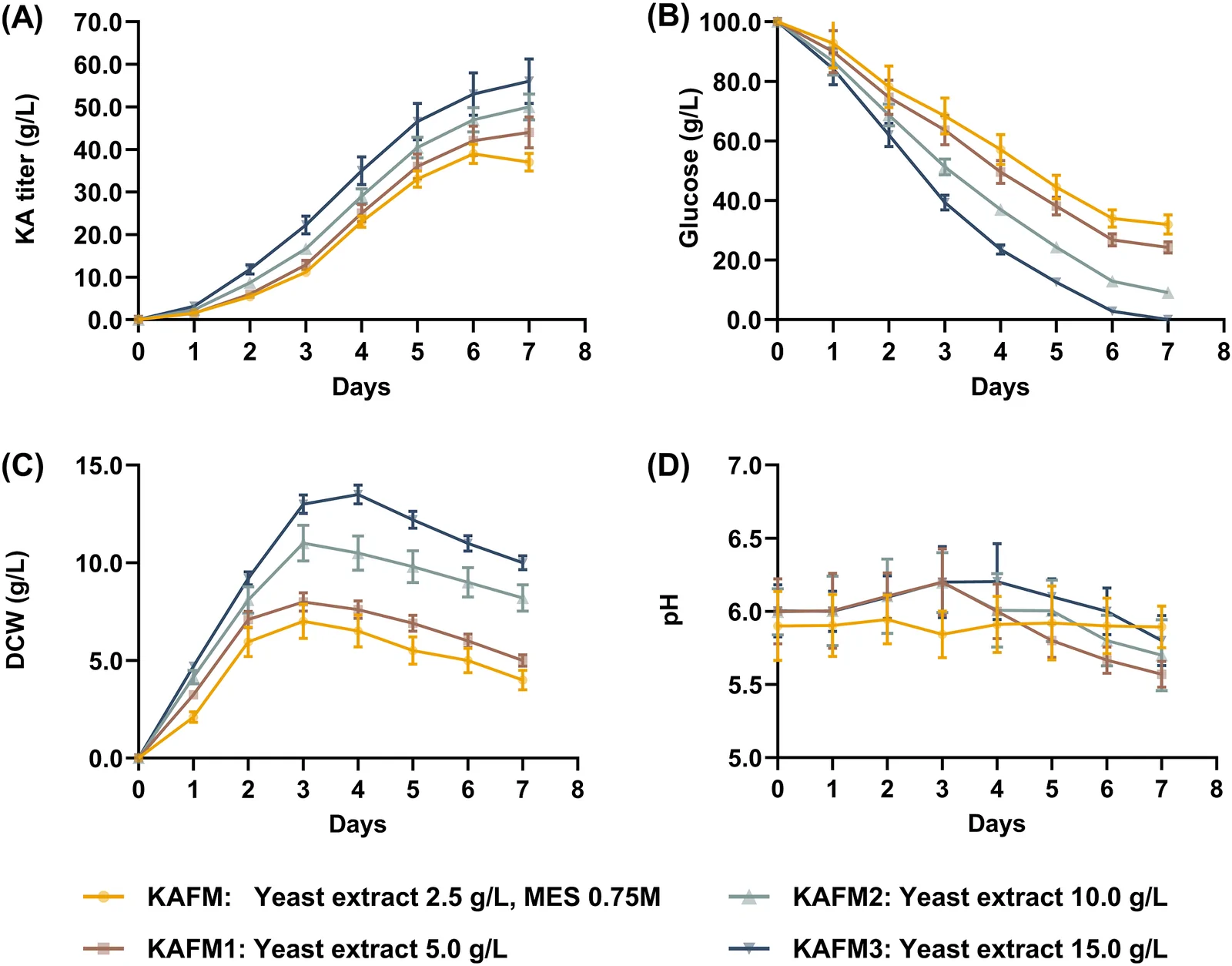
Bioreactor-scale evaluation of KA production by *A. niger* VHb-R in a 2-L stirred-tank fermenter. (A) KA titer, (B) residual glucose concentration, (C) dry cell weight (DCW), and (D) pH during fermentation. Media compared: original formulation (100 g/L glucose, 2.5 g/L yeast extract, 0.75 M MES) and MES-free media containing 5, 10, or 15 g/L yeast extract. Data are mean ± SD (n = 3). Two-way ANOVA. (∗p < 0.05, ∗∗p < 0.01, ∗∗∗p < 0.001, ∗∗∗∗p < 0.0001).

Finally, the optimized medium formulation was validated in a 2 L bioreactor to assess the scalability of the process. KA production was compared across four conditions: the original medium (2.5 g/L yeast extract with MES) and modified media containing 5, 10, or 15 g/L yeast extract without MES. In this scale-up, KA titers increased from 44.0 g/L at 5 g/L yeast extract to 56.0 g/L at 15 g/L, whereas the original formulation yielded only 37.0 g/L (Fig. 7A). These results highlight the beneficial effects of both increased nitrogen availability and the elimination of MES on KA production. Peak biomass levels ranged from 8.0 to 13.5 g/L (Fig. 7C), increasing with higher nitrogen concentrations, suggesting a positive correlation between biomass accumulation and KA production at the scale tested. In all groups, biomass gradually increased over the first 3 to 4 days of fermentation, followed by a steady decline. Notably, KA synthesis continued during the biomass decline phase, indicating that KA production was not strictly dependent on active cell growth and may persist during the transition to stationary phase. Residual glucose was consumed more rapidly in the bioreactor than in shake flasks. In the 15 g/L group, glucose was fully depleted by the end of fermentation, suggesting elevated metabolic activity under high-nitrogen conditions (Fig. 7B). The KA yields remained consistent across all groups, ranging from 0.54 to 0.58 g kA/g glucose.

The process demonstrated robust performance at both shake flask and 5 L bioreactor scales. In shake flasks, the maximum KA titer reached 50 g/L, with a yield of 0.61 g/g and a volumetric productivity of 7.1 g/L/day. Upon scale-up to the 5 L bioreactor, the maximum titer increased to 56 g/L, while the yield remained high at 0.58 g/g, and the volumetric productivity slightly improved to 8.0 g/L/day. Compared to literature-reported values (Table S3), the yield and volumetric productivity achieved in this study represent a relatively high level, exceeding the majority of previously documented strains, which typically exhibit yields in the range of 0.2–0.6 g/g and volumetric productivities below 8.0 g/L/day. However, comprehensive comparisons are often hindered by incomplete datasets in many reports, where not all three key performance indicators—titer, yield, and volumetric productivity—are simultaneously provided. Among the studies that do report all three metrics, only two strains exceed our results in all aspects: *A. oryzae* isolate Ao-4 (139.24 g/L, >0.70 g/g, 13.92 g/L/day) and isolate AR-47 (96.5 g/L, 0.60 g/g, 19.3 g/L/day) [19,33]. It is important to note that these strains were obtained via mutagenesis-based random screening, whereas our host was rationally designed through targeted metabolic engineering, which offers greater reproducibility and scalability for future industrial implementation.

Moreover, the scale-up from shake flasks to a 5L bioreactor only resulted in a 12% increase in the maximum KA titer, indicating that there is still room for optimization in oxygen transfer, mixing efficiency, and feeding strategies. Future studies could focus on using fed-batch fermentation strategies and optimizing critical operational parameters (e.g., dissolved oxygen control, feeding timing) to further improve production efficiency and scalability. In addition, several advanced fermentation strategies have been reported to significantly enhance KA production and may serve as valuable references for further optimization [[34], [35], [36], [37]]. These include resuspended cell systems, repeated-batch fermentation with cell retention, immobilized-cell systems, and membrane-surface liquid culture. These approaches improve production by enhancing oxygen transfer, extending production cycles, or stabilizing cell performance, and thus offer promising directions for improving the efficiency of engineered microbial systems. These findings provide essential references for industrial-scale production and valuable data for understanding the metabolic characteristics of this strain. Collectively, the findings of this study not only establish a solid foundation for industrial-scale KA production using metabolically engineered *A*. *niger*, but also contribute valuable insights into the metabolic characteristics and optimization potential of this novel microbial chassis.

## CRediT authorship contribution statement

**Jiao Liu:** Writing – review & editing, Writing – original draft, Validation, Methodology, Investigation, Data curation. **Bingbing Xie:** Writing – original draft, Investigation. **Fengna Gao:** Writing – original draft, Investigation. **Jing Kang:** Writing – original draft, Investigation. **Hao Liu:** Writing – review & editing, Resources, Project administration, Funding acquisition.

## Declaration of generative AI in scientific writing

The authors used generative AI and AI-assisted technologies only for language editing and improving readability; these tools did not contribute to the scientific content, data analysis, or interpretation.

## Declaration of competing interest

The authors declare that they have no known competing financial interests or personal relationships that could have appeared to influence the work reported in this paper.

## Data Availability

All data are publicly available.
