# Supplementary material for: Engineered Aspergillus niger enables high-yield heterologous kojic acid production
Source: Synth Syst Biotechnol. 2026 Mar 22;13:468–79. doi: 10.1016/j.synbio.2026.03.003 (PMC13494394; doi:10.1016/j.synbio.2026.03.003)
Supplement: Supplementary file 1 [file mmc1.docx]

**Supporting Information**

**Engineered *Aspergillus niger* enables high-yield heterologous kojic acid production**

**AUTHOR NAMES:**

Jiao Liu, Bingbing Xie, Fengna Gao, Jing Kang, Hao Liu*

**AUTHOR ADDRESS:**

Key Laboratory of industrial Fermentation Microbiology, Ministry of Education, Tianjin Key Laboratory of Industrial Microbiology, College of Biotechnology, Tianjin University of Science and Technology, Tianjin 300457, China.

^*^ **Corresponding Author:** *E-mail address*: liuhao@tust.edu.cn (H. Liu)

**List of Supporting Information**

Table S1 The *A*. *niger* strains and Plasmids used in this study

Table S2 Primers used in this study

Table S3 Literature review of prior studies on KA fermentation by various *Aspergillus* species

Figure S1 Growth inhibition of *Aspergillus niger* S834 and *Aspergillus oryzae* NRRL3488 on Potato Dextrose Agar plates supplemented with kojic acid at concentrations (conc.) of 0-50 g/L after incubation at 28°C for 5 days.

**Table S1 The *A*. *niger*** **strains and Plasmids used in this study**

| **Strains/ Plasmids** | **Characteristics** | | **Source** |
| --- | --- | --- | --- |
| **Strains** | |  |  |
| S834 | | *Tet*-*On*::*cre*, Δ*oahA*, Δ*cexA*, hyg^s^ | Lab stock^1^ |
| S1991 | | *PgpdA*::*kojA*::*TtrpC*, hygr | Lab stock ^1^ |
| KH-R | | *PgpdA*::*kojA*-*his*::*TtrpC*, hyg^r^ | This study |
| KH-S | | *PgpdA*::*kojA*-*his*::*TtrpC*, hyg^s^ | This study |
| 2KH-R | | 2× *PgpdA*::*kojA*-*his*::*TtrpC*, hyg^r^ | This study |
| 2KH-S | | 2× *PgpdA*::*kojA*-*his*::*TtrpC*, hyg^s^ | This study |
| 3KH-R | | 3× *PgpdA*::*kojA*-*his*::*TtrpC*, hyg^r^ | This study |
| 3KH-S | | 3× *PgpdA*::*kojA*-*his*::*TtrpC*, hyg^s^ | This study |
| ΔAngkojT-R | | *PgpdA*::*kojA*-*his*::*TtrpC*, Δ*AngkojT*, hyg^r^ | This study |
| AngkojT-R | | 3× *PgpdA*::*kojA*-*his*::*TtrpC*, *PgpdA*::*AngkojT*::*TtrpC*, hyg^r^ | This study |
| AogkojT-R | | 3× *PgpdA*::*kojA*-*his*::*TtrpC*, *PgpdA*::*AokojT*::*TtrpC*, hyg^r^ | This study |
| AogkojT-S | | 3× *PgpdA*::*kojA*-*his*::*TtrpC*, *PgpdA*::*AokojT*::*TtrpC*, hyg^s^ | This study |
| ΔnrkC-R | | 3× *PgpdA*::*kojA*-*his*::*TtrpC*, *PgpdA*::*AokojT*::*TtrpC*, Δ*nrkC*, hyg^r^ | This study |
| ΔnrkC-S | | 3× *PgpdA*::*kojA*-*his*::*TtrpC*, *PgpdA*::*AokojT*::*TtrpC*, Δ*nrkC*, hyg^s^ | This study |
| ΔLaeA-R | | 3× *PgpdA*::*kojA*-*his*::*TtrpC*, *PgpdA*::*AokojT*::*TtrpC*, Δ*nrkC*, Δ*laeA*, hyg^r^ | This study |
| LaeA-R | | 3× *PgpdA*::*kojA*-*his*::*TtrpC*, *PgpdA*::*AokojT*::*TtrpC*, Δ*nrkC*, *PgpdA*::*laeA*::*TtrpC*, hyg*^r^* | This study |
| LaeA-S | | 3× *PgpdA*::*kojA*-*his*::*TtrpC*, *PgpdA*::*AokojT*::*TtrpC*, Δ*nrkC*, *PgpdA*::*laeA*::*TtrpC*, hyg^s^ | This study |
| VHb-R | | 3× *PgpdA*::*kojA*-*his*::*TtrpC*, *PgpdA*::*AokojT*::*TtrpC*, Δ*nrkC*, *PgpdA*::*laeA*::*TtrpC*, *PgpdA*::*vgb*::*TtrpC*, hyg^r^ | This study |
| **Plasmids** | |  |  |
| pLH454 | | *PgpdA*, *TtrpC*, *lox*P*-hph-lox*P, hyg^r^, kan^r^ | Lab stock **^2^** |
| pLH594 | | *PgpdA*, *TtrpC*, *lox*P*-hph-lox*P, hyg^r^, ppt^r^, kan^r^ | Lab stock **^3^** |
| pLH1497 | | *ASPNIDRAFT_186610*::*lox*P-*hph*-*lox*P, hyg^r^, ppt^r^, kan^r^ | Lab stock **^1^** |
| pLH1737 | | *ASPNIDRAFT_43217*::*lox*P-*hph*-*lox*P, hyg^r^, ppt^r^, kan^r^ | Lab stock **^1^** |
| pLHVHB | | *PgpdA*:: *vgb*::*TtrpC*, *lox*P*-hph-lox*P, hyg^r^, kan^r^ | Lab stock **^4^** |
| pKH | | *PgpdA*:: *kojA*-*his*::*TtrpC*, *lox*P*-hph-lox*P, hyg^r^, kan^r^ | This study |
| pANKT | | *PgpdA*:: *AngkojT*::*TtrpC*, *lox*P*-hph-lox*P, hyg^r^, kan^r^ | This study |
| pAOKT | | *PgpdA*:: *AokojT*::*TtrpC*, *lox*P*-hph-lox*P, hyg^r^, kan^r^ | This study |
| pLAEA | | *PgpdA*::*laeA*::*TtrpC*, *lox*P*-hph-lox*P, hyg^r^, kan^r^ | This study |
| pDLAEA | | *laeA*::*lox*P-*hph*-*lox*P, hyg^r^, ppt^r^, kan^r^ | This study |

hyg^r^, hygromycin B resistance; hyg^s^, hygromycin B sensitive; ppt^r^, phosphinothricin resistance; kan^r^, kanamycin resistance

**Table S2 Primers used in this study**

| Primer and Purpose | Sequence（5'-3'） |
| --- | --- |
| pKH construction | |
| KH-F | CACATCTAAACAATGGAATTCCGTGTCGCGACACAGCTAA |
| KH-R | ACGTTAAGTGGATCCTTAGTGGTGGTGGTGGTGGTGGTTTGCAGTCACTAGTGAAACAT |
| pANKT construction | |
| ANKT-F | CACATCTAAACAATGATGCAGTCCTTCCTTC |
| ANKT-R | ACGTTAAGTGGATCCCTATGCTGCGGCAGACTTG |
| pAOKT construction | |
| AOKT-F | CACATCTAAACAATGATGCAGTCCTTCCGCC |
| AOKT-R | ACGTTAAGTGGATCCTCAGGGGGTCTTTTTATTCCGC |
| pLAEA construction | |
| LAEA-F | CACATCTAAACAATGATGGGCTCAATCGCCCCTG |
| LAEA-R | ACGTTAAGTGGATCCCTACATCGGCTTCTGCGCC |
| pDLAEA construction | |
| laeA-UF | GCTCCGTAACACCCAGAATTC TATAGAGACCGTCGATTCCTTAATC |
| laeA-UR | TTATACGAACGGTAGGATCC CCCCACAAGAAAGCCCG |
| laeA-DF | GCTATACGAACGGTATCTAGA CGCCATGTATGGCGCACC |
| laeA-DR | GTGCCAAGCTTGCATGCCTGCAG GATATACAGAAAGCCACATACTTCAGG |
| *kojA-his* overexpression verification | |
| KH-P1 | ATGGAATTCCGTGTCGCGACACAGCTAA |
| hyg-P2 | TTAACTGGTTCCCGGTCGGC |
| *AngkojT* overexpression verification | |
| ANKT-P1 | ATGCAGTCCTTCCTTC |
| hyg-P2 | TTAACTGGTTCCCGGTCGGC |
| *AokojT* overexpression verification | |
| AOKT-P1 | ATGCAGTCCTTCCGCC |
| hyg-P2 | TTAACTGGTTCCCGGTCGGC |
| *laeA* overexpression verification | |
| LAEA-P1 | ATGGGCTCAATCGCCCCTG |
| hyg-P2 | TTAACTGGTTCCCGGTCGGC |
| *vgb* overexpression verification |  |
| VHb-P1 | ATGTTAGATCAACAAACTATTAATATTATTAAAG |
| hyg-P2 | TTAACTGGTTCCCGGTCGGC |
| *AngkojT* deletion verification | |
| ANKT-DP1 | GCTACTGGATGGGTCTTACG |
| ANKT-DP2 | GCCGAGCGTCTTCATAGTC |
| ANKT-DP3 | GTATCTCCCTACTGATGACGCC |
| ANKT-DP4 | GCCGTGGGAATTAATGTGGG |
| *nrkC* deletion verification | |
| nrkC-DP1 | CGAGAATATCAATTTGCCTCCG |
| nrkC-DP2 | CGCGGAAAGCATTGCTTC |
| Gene deletion verification (corresponding to the internal region of *hph*) | |
| P641 | CAATATCAGTTAACGTCGAC |
| P642 | GGAACCAGTTAACGTCGAAT |
| *hph* elimination verification |  |
| hph-F | GTGGAGGTCAACAATGAATGCC |
| hph-R | GTCGGTTTCCACTATCGGC |
| *kojA* qRT-PCR |  |
| qPCR-kojA-F | CTTGACACACGGCAGTTACAC |
| qPCR-kojA-R | GTTACAACGTTGCTCACCTTGC |
| beta-actin qRT-PCR | |
| qPCR-actA-F | TCCTCACCCTCAGATACCC |
| qPCR-actA-R | CACCGTCACCAGAGTCCA |

**Table S3 Literature review of prior studies on KA fermentation by various *Aspergillus* species**

| **Strains** | **Cultivation method** | **pH** | **Temperature**  **(°C)** | **Bior. Type** | **Nitrogen**  **(g/L)** | **Carbon**  **(g/L)** | **KA titer** **(g/L)** | **Yield**  **(g/g)** | **Productivity**  **(g/L/d)** | **References** |
| --- | --- | --- | --- | --- | --- | --- | --- | --- | --- | --- |
| *A. candidus* ATCC 44054 | SBC (Static) | 6.5 | 25 | Shake flask | YE (20) | Sucrose (200) | 59.0 | NCAL | 4.92 | **5** |
| *A. flavus* OL314748 (ASU45) | ---- | ---- | 28 | ---- | YE (5) | Glucose (150) | 81.59 | NCAL | 8.16 | **6** |
| *A. oryzae* ASU44 and  *A. favus* ASU45 | ---- | ---- | 28 | ---- | YE (5) | Glucose (150) | 114.28 | NCAL | 11.43 | **7** |
| *A. flavus* | SBC (Static) | 6.0 | 30 | Shake flask | YE (5) | Sucrose (70) | 29.60 | NCAL | 2.47 | **8** |
| *A. flavus* | SBC (Shaken) | 4.5 | 30 | Shake flask | YE (5) | Glucose (100) | 16.00 | NCAL | 0.80 | **9** |
| *A. flavus* isolate 7 | SBC (Shaken) | 3.0 | 28 | Fermenter | YE (5) | Glucose (100) | 53.50 | NCAL | 6.68 | **10** |
|  |  |  |  | Shake flask |  |  | 34.40 | NCAL | 3.44 |  |
| *A. flavus* isolate 44-1 | SBC (Shaken) | 3.5 | 30 | Fermenter | YE (5) | Glucose (100) | 28.90 | 0.29 | 0.13 | **11** |
| *A. flavus* isolate 44-1 | SBC (Shaken) | 3.0 | 30 | Fermenter | YE (5) | Glucose (100) | 36.50 | 0.37 | 3.31 | **12** |
|  |  |  |  | Shake flask | YE (5) |  | 32.50 | 0.33 | 1.38 |  |
|  |  |  |  | RCS. F. | YE (0) |  | 48.10 | 0.481 | 3.72 |  |
| *A. flavus* isolate 44-1 | SBC (Shaken) | 3.0 | 30 | Shake flask | YE (5) | Glucose (100) | 39.90 | 0.99 | 1.99 | **13** |
|  |  |  |  | RCS. SF. | YE (0) | Glucose (150) | 42.750 | 0.29 | 1.94 |  |
| *A. flavus* isolate 44-1 | SBC (Shaken) | 3.0 | 30 | Fermenter | YE (5) | Sucrose (150) | 40.20 | 0.27 | 3.65 | **14** |
|  |  |  |  | Shake flask |  | Glucose (100) | 25.80 | 0.26 | 1.30 |  |
| *A. flavus* isolate 44-1 | SBC (Shaken) | 3.5 | 30 | RCS. SF. | YE (0) | Glucose (100) | 45.30 | 0.45 | 2.06 | **15** |
| *A. flavus* isolate 44-1 | SBC (Shaken) | 3.0 | 30 | Fermenter | YE (5) | Glucose (100) | 44.93 | 0.46 | 2.24 | **16** |
| *A. flavus* isolate B | SBC (Static) | 6.5 | 30 | Fermenter | YE (20) | Glucose (200) | 80.20 | NCAL | 10.20 | **17** |
| *A. flavus* isolate No.3538 | SC (Static) | - | 25 | Shake flask | NH_4_NO_3_ | Dextrose (220) | 17.90 | 0.44 | 2.56 | **18** |
| *A. flavus* IOC 3974 | SBC (Shaken) | 5.5 | 28 | Shake flask | NaNO_3_ | Sucrose (120) | 26.00 | 0.22 | 1.73 | **19** |
| *A. flavus* | SBC (Shaken) | 6.5 | 30 | Shake flask | NH_4_NO_3_ | Sucrose (100) and Fructose (100) | 30.00 | NCAL | 3.00 | **20** |
| *A. flavus* | SBC (Shaken) | 3.0 | 30 | Shake flask | YE (2) | Glucose (100) | 31.50 | 0.36 | 2.42 | **21** |
| *A. oryzae* T58 | SBC (Shaken) | -- | 33 | Shake flask | Tryptone (4) | Dextrin (90) | 32.50 | NCAL | 6.50 | **22** |
| *A. oryzae* isolate 1034 | SC (Static) | 4.5 | 28 | Shake flask | YE (5) | Glucose (60) | 79.30 | EMTY | 7.20 | **23** |
| *A. oryzae* isolate Ao-4 | SBC (Shaken) | 3.0 | 28 | Shake flask | YE (6) | Glucose (200) | 139.24 | >0.70 | 13.92 | **24** |
| *A. oryzae* isolate AR-47 | SBC (Shaken) | 4.0 | 30 | Shake flask | YE (8) | Glucose (160) | 96.50 | 0.60 | 19.30 | **25** |
| *A. oryzae* BCRC 30010 | SBC (Shaken) | 3.0 | 30 | IC.SF | YE (1) | Glucose (100) | 83.47 | 0.36 | 3.09 | **26** |
|  |  |  |  | Shake flask | YE (5) | Glucose (100) | 35.24 | NCAL | NCAL |  |
| *A. oryzae* isolate M3B9 | SBC (Shaken) | 5.0 | 30 | Shake flask | YE (2.5) | Sucrose (100) | 41.00 | NCAL | 5.10 | **27** |
|  |  |  |  | Fermenter (5L) | YE (2.5) | Sucrose (100) | 52.00 | 0.26 | 3.50 |  |
|  |  |  |  | RB.F | YE (2.5) | Sucrose (100) | 355.47 | NCAL | 4.40 |  |
| *A. oryzae* isolate M866 | SBC (Shaken) | 6.0 | 30 | Shake flask | YE (2.5) | Glucose (100) | 40.20 | 0.44 | 5.02 | **28** |
| *A. oryzae* isolate MK-107-39 | SBC (Shaken) | 4.0 | 30 | Fermenter (600 L) | Wheat Germ (Feed) | Glucose (Feed) | 117.60 | 0.43 | 13.07 | **29** |
|  |  |  |  | Shake flask | Peptone (7) | Glucose (100) | 28.00 | 0.30 | 7.00 |  |
| *A. oryzae* isolate No. 69 | SBC (Shaken) | 4.0 | 30 | Shake flask | Peptone (5) | Glucose (100) | 24.00 | 0.26 | 3.43 | **30** |
| *A. oryzae* NRRL 484 | SBC (Shaken) | 6.0 | 30 | IC.SF | YE (Feed) | Glucose (Feed) | 80.00 | 0.4 | 3.70 | **31** |
| *A. oryzae* NRRL 484 | SBC (Shaken) | 6.0 | 30 | Bath MSLC | YE (2.5) | Glucose (100) | 29.00 | 0.45 | 2.90 | **32** |
|  |  |  |  | fed-batch MSLC |  |  | >100.00 | 0.56 | 5.00 |  |
|  |  |  |  | Shake flask |  |  | 20.60 | 0.51 | 1.60 |  |
| *A. oryzae* NRRL 484 | SBC (Shaken) | 6.0 | 30 | Bath MSLC | YE (1.0) | Glucose (200) | 60.00 | 0.30 | 2.22 | **33** |
|  |  |  |  | Bath MSLC | YE (1.0) | Glucose (100) | 45.00 | 0.55 | 3.30 |  |
|  |  |  |  | Continuous MSLC | YE (1.0) | Glucose (100) | 47.00 | 0.70 | 1.8 |  |
|  |  |  |  | Shake flask | YE (1.0) | Glucose (100) | 24.00 | 0.31 | 1.5 |  |
| *A. oryzae* NSPID1/pIS1-kojR | SBC (Shaken) | 6.0 | 30 | Shake flask | YE (2.5) | Glucose (80) | 26.40 | NCAL | 1.89 | **34** |
| *A. oryzae var. effusus* NRC14 | SC (Static) | 4.0 | 28 | Shake flask | NH_4_NO_3_ (1.13) | Glucose (100) | 42.00 | 0.626 | 2.33 | **35** |
| *A. oryzae var. effusus* NRC14 | SC (Static) | 4.0 | 30 | Shake flask | NH_4_NO_3_ (1.13) | Glucose (100) | 50.00 | 0.85 | 4.17 | **36** |
| *A. oryzae* | SBC (-) | 4.5 | 30 | Shake flask | NaNO_3_ (5) | Molasses (50) | 72.43 | NCAL | 10.35 | **37** |
| *A. parasiticus* | SBC (Shaken) | 5.0 | 30 | Shake flask | YE (10) | Glucose (60) | 34.38 | NCAL | 3.44 | **38** |
| *A. sojae* SSC-3 | SBC (Shaken) | 6.4 | 28 | Fermenter | YE (5) | Sucrose (20) | 18.20 | EMTY | 3.64 | **39** |
| *A. terreus* C5-10 mutant | SBC (Static) | 2.1 | 30 | Shake flask | YE (1.0) | Glucose (98.4) | 109.00 | NCAL | NCAL | **40** |
| *Petromyces flavus* isolate No. 64 | SBC (Shaken) | 3.0 | 28 | Shake flask | YE (5) | Glucose (100) | 36.75 | NCAL | NCAL | **41** |

Bior. Type: bioreactor type; EMTY: Exceed the maximum theoretical yield; IC.SF: Immobilized cell in shake flask; NCAL: Due to insufficient data provided in the literature, it is not possible to calculate the parameter Yield; SBC: Submerged culture; SC: Surface culture; RCS. F.: Resuspended cell system in fermentor; RCS. SF.: Resuspended cell system in shake flask; RB.F: Repeated-batch production of kojic acid in a cell-retention fermenter; MSLC：Membrane-surface liquid culture; YE: Yeast extract;

**REFERENCES**

(1) Wu, L.; Zhang, L.; Li, X.; Lv, R.; Cao, W.; Gao, W.; Liu, J.; Xie, Z.; Liu, H. Effective production of kojic acid in engineered *Aspergillus niger*. *Microb. Cell Fact.* **2023**, *22* (1), 40. DOI: 10.1186/s12934-023-02038-w.

(2) Xu, Y.; Shan, L.; Zhou, Y.; Xie, Z.; Ball, A. S.; Cao, W.; Liu, H. Development of a Cre-*lox*P-based genetic system in *Aspergillus niger* ATCC 1015 and its application to construction of efficient organic acid-producing cell factories. *Appl. Microbiol. Biotechnol.* **2019**, *103* (19), 8105-8114. DOI: 10.1007/s00253-019-10054-3.

(3) Cao, W.; Yan, L.; Li, M.; Liu, X.; Xu, Y.; Xie, Z.; Liu, H. Identification and engineering a C4-dicarboxylate transporter for im-provement of malic acid production in *Aspergillus niger*. *Appl. Microbiol. Biotechnol.* **2020**, *104* (22), 9773-9783. DOI: 10.1007/s00253-020-10932-1.

(4) Liu, J.; Zhang, Q.; Liang, X.; Zhang, R.; Huang, X.; Zhang, S.; Xie, Z.; Gao, W.; Liu, H. Improving glucose oxidase catalysis in *Aspergillus niger* via Vitreoscilla hemoglobin fusion protein. *Appl. Microbiol. Biotechnol.* **2024**, *108* (1), 48. DOI: 10.1007/s00253-023-12931-4 From NLM.

(5) Wei, C. I.; Huang, T. S.; Chen, J. S.; Marshall, M. R.; Chung, K. T. Production of kojic acid by *Aspergillus candidus* in three culture media. *J. Food Prot.* **1991**, *54* (7), 546-548.

(6) Mahmoud, G. A.; Abdel Shakor, A. B.; Kamal-Eldin, N. A.; Zohri, A. A. Production of kojic acid by *Aspergillus flavus* OL314748 using box-Behnken statistical design and its antibacterial and anticancer applications using molecular docking technique. *BMC Microbiol.* **2024**, *24* (1), 140. DOI: 10.1186/s12866-024-03289-2 From NLM.

(7) Kamal-Eldin, N. A.; Zohri, A. N. A.; Mahmoud, A. E. Optimization of kojic acid production by *Aspergillus* coculture and it's application as anti-browning agent. *Bull. Pharm. Sci.* **2023**, *46* (2), 1269-1283.

(8) H., A.-S. F. K.; M., A. A. H. Optimum conditions for the production of kojic acid from the fungus *Aspergillus flavus*. *Turk. J. Physiother. Rehabil.* **2021**, *32* (3), 10013-10018.

(9) Badar, R.; Yaqoob, S.; Ahmed, A.; Shaoor, Q. U. A. Screening and optimization of submerged fermentation of aspergillus species for kojic acid production. *Res. Sq.* **2021**, *PREPRINT*. DOI: 10.21203/rs.3.rs-334019/v1.

(10) El-Kady, I. A.; Zohri, A. N. A.; Hamed, S. R. Kojic acid production from agro-industrial by-products using fungi. *Biotechnol. Res. Int.* **2014**, *2014*, 642385.

(11) Ariff, A. B.; Salleh, M. S.; Ghani, B.; Hassan, M. A.; Karim, M. I. A. Aeration and yeast extract requirements for kojic acid production by *Aspergillus flavus* link. *Enzyme Microb. Technol.* **1996**, *19* (7), 545-550.

(12) Ariff, A. B.; Rosfarizan, M.; Herng, L. S.; Madihah, S.; Karim, M. I. A. Kinetics and modelling of kojic acid production by *Aspergillus flavus* Link in batch fermentation and resuspended mycelial system. *World J. Microbiol. Biotechnol.* **1997**, *13* (2), 195-201.

(13) Rosfarizan, M.; Ariff, A. B. Kinetics of kojic acid fermentation by *Aspergillus flavus* using different types and concentrations of carbon and nitrogen sources. *J. Ind. Microbiol. Biotechnol.* **2000**, *25* (1), 20-24.

(14) Rosfarizan, M.; Ariff, A. B. Kinetics of kojic acid fermentation by *Aspergillus flavus* link S44-1 using sucrose as a carbon source under different pH conditions. *Biotechnol. Bioprocess Eng.* **2006**, *11* (1), 72-79.

(15) Mohamad, R.; Ariff, A. B. Biotransformation of various carbon sources to kojic acid by cell-bound enzyme system of *A. flavus* Link 44-1. *Biochem. Eng. J.* **2007**, *35* (2), 203-209.

(16) Suhaili, N.; Tan, J. S.; Mohamed, M. S.; Halim, M.; Ariff, A. B. Effects of dual impeller system of Rushton turbine, concave disk turbine and their combinations on the performance of kojic acid fermentation by *Aspergillus flavus* Link 44-1. *Asia-Pac. J. Chem. Eng.* **2015**, *10* (1), 65-74.

(17) Bajpai, P.; Agrawala, P. K.; Vishwanathan, L. Production of kojic acid by resuspended mycelia of *Aspergillus flavus*. *Can. J. Microbiol.* **1982**, *28* (12), 1340-1346.

(18) May, O. E.; Moyer, A. J.; Wells, P. A.; Herrick, H. T. The production of kojic acid by *Aspergillus flavus*. *J. Am. Chem. Soc.* **1931**, *53* (2), 774-782. DOI: 10.1021/ja01353a050.

(19) Ferreira, N. R.; Sarquis, M. I.; Alves, C. N.; Santos, A. S. Biotransformation of sucrose into 5-hydroxy-2-hydroxymethyl-γ -pirone by *Aspergillus flavus*. *An. Acad. Bras. Cienc.* **2011**, *82* (3), 569-576.

(20) Fadhil, K.; Majeed, M.; Ahmed, M. Electronic study of fresh enzyme complexes of antifungal drugs-P450 and *Aspergillus* kojic acid biosynthesis. W: w saccharose flavus: fructose as a substratum. *Afr. Health Res. Org.* **2019**, *22*, 65-72. DOI: 10.36295/ASRO.2019.22048.

(21) Sanjotha, G.; Shivasharana, C. T.; Manawadi, S. I. An in vitro approach for evaluating antimicrobial activity and production of kojic acid by *Aspergillus flavus* isolated from Karwar region. *J. Pure. Appl. Microbiol.* **2019**, *13* (4), 2261-2272. DOI: <https://doi.org/10.22207/JPAM.13.4.40>.

(22) Zhang, X.; Guo, R.; Bi, F.; Chen, Y.; Xue, X.; Wang, D. Overexpression of *kojR* and the entire koj gene cluster affect the kojic acid synthesis in *Aspergillus oryzae* 3.042. *Gene* **2024**, *892*, 147852. DOI: 10.1016/j.gene.2023.147852 From NLM.

(23) Rasmey, A. H.; Abdel-Kareem, M. Optimization of culture conditions for kojic acid production in surface fermentation by *Aspergillus oryzae* isolated from wheat grains. *Bull. Pharm. Sci. Assiut* **2021**. DOI: 10.21608/BFSA.2021.174146.

(24) Mahmoud, A. E.; Zohri, A. N. A. Amedment stable kojic acid produced by non-toxinogenic *Aspergillus oryzae* using five levels central composite design of response surface methodology. *J. Microbiol. Biotechnol. Food Sci.* **2021**, *10* (6), e2683. DOI: 10.15414/jmbfs.2683.

(25) Feng, W.; Liang, J.; Wang, B.; Chen, J. Improvement of kojic acid production in *Aspergillus oryzae* AR-47 mutant strain by combined mutagenesis. *Bioprocess Biosyst. Eng.* **2019**, *42* (5), 753-761.

(26) Liu, J.; Yu, T.; Lin, S.; Hsu, R. J.; Hsu, K. D.; Cheng, K. Evaluation of kojic acid production in a repeated-batch PCS biofilm reactor. *J. Biotechnol.* **2016**, *218*, 41-48. DOI: 10.1016/j.jbiotec.2015.11.023. Epub 2015 Dec 3.

(27) Wan, H. M.; Chen, C. C.; Giridhar, R.; Chang, T. S.; Wu, W. T. Repeated-batch production of kojic acid in a cell-retention fermenter using *Aspergillus oryzae* M3B9. *J. Ind. Microbiol. Biotechnol.* **2005**, *32* (6), 227-233.

(28) Yan, S.; Tang, H.; Wang, S.; Xu, L.; Liu, H.; Guo, Y.; Yao, J. Improvement of kojic acid production in *Aspergillus oryzae* B008 mutant strain and its uses in fermentation of concentrated corn stalk hydrolysate. *Bioprocess Biosyst. Eng.* **2014**, *37* (6), 1095-1103. DOI: 10.1007/s00449-013-1081-5 From NLM.

(29) Futamura, T.; Okabe, M.; Tamura, T.; Toda, K.; Matsunobu, T.; Park, Y. S. Improvement of production of Kojic acid by a mutant strain *Aspergillus oryzae*, MK107-39. *J. Biosci. Bioeng.* **2001**, *91* (3), 272-276.

(30) Kitada, M.; Ueyama, H.; Suzuki, E.; Fukimbara, T. Studies on kojic acid fermentation : (I) cultural conditions in submerged Culture. *J. Ferment. Technol.* **1967**, *45* (12), 1101-1107.

(31) Kwak, M. Y.; Rhee, J. S. Controlled mycelial growth for kojic acid production using Ca-alginate-immobilized fungal cells. *Appl. Microbiol. Biotechnol.* **1992**, *36* (5), 578-583.

(32) Ogawa, A.; Wakisaka, Y.; Tanaka, T.; Sakiyama, T.; Nakanishi, K. Production of kojic acid by membrane-surface liquid culture of *Aspergillus oryzae* NRRL484. *J. ferment. bioeng* **1995**, *80* (1), 41-45.

(33) Wakisaka, Y.; Segawa, T.; Imamura, K.; Sakiyama, T.; Nakanishi, K. Development of a cylindrical apparatus for membrane-surface liquid culture and production of kojic acid using *Aspergillus oryzae* NRRL484. *J. Ferment. Bioeng.* **1998**, *85* (5), 488-494.

(34) Yamada, R.; Yoshie, T.; Wakai, S.; Asai-Nakashima, N.; Okazaki, F.; Ogino, C.; Hisada, H.; Tsutsumi, H.; Hata, Y.; Kondo, A. *Aspergillus oryzae*-based cell factory for direct kojic acid production from cellulose. *Microb. Cell Fact.* **2014**, *13*, 71. DOI: 10.1186/1475-2859-13-71 From NLM.

(35) Hazzaa, M.; Hassan, H.; Ibrahim, E. High production of KA crystals by isolated *Aspergillus oryzae var. effusus* NRC14. *J. Appl. Sci. Res.* **2013**, *9* (2013), 1714-1723.

(36) Hassan, H.; Saad, A.; Hazzaa, M.; Ibrahim, E. Optimization study for the production of kojic acid crystals by *Aspergillus oryzae var. effusus* nrc 14 isolate. *Int. J. Curr. Microbiol. Appl. Sci.* **2014**, *3* (3), 133-142.

(37) Ibrahim, S.; Saleh, N. M. Study of the optimal nutritional conditions for the production of kojic acid from the local isolation of the Aspergillus oryzae. *Plant Archives* **2018**, *18*, 1801-1808.

(38) El-Aasar, S. Cultural conditions studies on kojic acid production by *Aspergillus parasiticus*. *Int. J. Agric. Biol.* **2006**, *8* (4), 468-473.

(39) Chib, S.; Dogra, A.; Nandi, U.; Saran, S. Consistent production of kojic acid from *Aspergillus sojae* SSC-3 isolated from rice husk. *Mol. Biol. Rep.* **2019**, *46* (6), 5995-6002. DOI: 10.1007/s11033-019-05035-8 From NLM.

(40) Shakibaie, M.; Ameri, A.; Ghazanfarian, R.; Adeli-Sardou, M.; Amirpour-Rostami, S.; Torkzadeh-Mahani, M.; Imani, M.; Forootanfar, H. Statistical optimization of kojic acid production by a UV-induced mutant strain of *Aspergillus terreus*. *Braz. J. Microbiol.* **2018**, *49* (4), 865-871. DOI: 10.1016/j.bjm.2018.03.009 From NLM.

(41) Moharram, A.; Seddek, A. Z., N. . Production of kojic acid by endophytic fungi isolated from medicinal plant in Egypt. *Int. Invent. J. Biochem. Bioinf.* **2015**, *3* (3), 28-31.

**Figure S1 Growth inhibition of *Aspergillus niger* S834 and *Aspergillus oryzae* NRRL3488 on Potato Dextrose Agar plates supplemented with kojic acid at concentrations (conc.) of 0-50 g/L after incubation at 28°C for 5 days.**
